# Integrated Single-Cell and Spatial Transcriptomics Analyses Delineate a *BAG3*-Associated Macrophage Program with Microenvironmental and Prognostic Relevance in Hepatocellular Carcinoma

**DOI:** 10.3390/genes17050562

**Published:** 2026-05-11

**Authors:** Ruixiang Zhang, Yifang Wei, Junda Yu, Yuansheng Li, Zuming You, Chenxi Xie, Siqi Xu, Jiyuan Zhou

**Affiliations:** 1 Department of Biostatistics, School of Public Health (State Key Laboratory of Multi-Organ Injury Prevention and Treatment, and Guangdong Provincial Key Laboratory of Tropical Disease Research), Southern Medical University, Guangzhou 510515, China; 2 Clinical Research Centre, School of Medicine, The Second Affiliated Hospital of South China University of Technology (Guangzhou First People’s Hospital), Guangzhou 510180, China

**Keywords:** hepatocellular carcinoma, tumor-associated macrophages, single-cell RNA sequencing, spatial transcriptomics, tumor microenvironment

## Abstract

**Background:** Tumor-associated macrophages (TAMs) are key components of the hepatocellular carcinoma (HCC) microenvironment, but their spatial heterogeneity remains incompletely characterized. We aimed to assess the biological and prognostic relevance of a *BAG3*-associated TAM program in HCC. **Methods:** Public single-cell RNA sequencing (scRNA-seq) datasets were analyzed to characterize TAM heterogeneity, and an integrated validation scRNA-seq dataset was used to assess reproducibility. Spatial transcriptomics was used to provide spatial context in a small treatment-exposed cohort. Pseudotime, regulatory network, and cell–cell communication analyses were performed to characterize state transitions and microenvironmental interactions. Survival modeling evaluated the prognostic relevance of the *BAG3*-associated program. **Results:** Five TAM subsets were identified, including MARCO^+^, MT^+^ RTM−, MMP9^+^, UBE2C^+^, and BAG3^+^ TAMs. Among them, BAG3^+^ TAMs, a less well-characterized subset, exhibited coordinated stress-adaptive, proteostasis-related, and matrix-remodeling programs that were reproduced in the validation dataset. Pseudotime analysis suggested a continuum of TAM states, with BAG3^+^ TAM stress-remodeling features enriched toward late pseudotime. Communication analysis centered on BAG3^+^ TAMs suggested crosstalk between inflammatory stress cues and angiogenic, stromal-remodeling, and immunomodulatory programs; this pattern was primarily supported by HBV-derived samples and recurrently involved the MIF–CD74 axis. Spatial mapping further supported BAG3^+^ TAM-enriched niches with elevated AP-1, EGR1, and NFKB1 activity. A *BAG3*-associated risk score derived from a 10-gene signature remained an independent prognostic factor for overall survival after clinical adjustment. **Conclusions:** These findings characterize a *BAG3*-associated TAM program with spatial, immunoregulatory, and prognostic relevance in HCC, and support its further evaluation in biomarker and mechanistic studies.

## 1. Introduction

Hepatocellular carcinoma (HCC), the most common primary liver cancer, is the third leading cause of cancer-related death worldwide, with advanced-stage disease showing a three-year overall survival rate below 40% [1,2]. HCC remains resistant to conventional therapies and tyrosine kinase inhibitors such as sorafenib [3,4,5], and responds poorly to PD-1/PD-L1 blockade. This limited efficacy largely reflects the profound heterogeneity of the HCC tumor microenvironment (TME) [6]. Among TME components, tumor-associated macrophages (TAMs) are the dominant innate immune population and are increasingly recognized as therapeutic targets, given that their high infiltration was consistently associated with poor prognosis in HCC [7]. TAMs promote tumor progression through metabolic reprogramming, angiogenesis, and T cell suppression [7,8]. Recent single-cell RNA sequencing (scRNA-seq) studies have revealed substantial TAM heterogeneity beyond the classical M1 (anti-tumor)/M2 (tumor-promoting) polarization framework [8,9,10] and identified macrophage-surface targets such as MARCO and TREM1 [11,12].

However, the functional and spatial heterogeneity of TAM states in native HCC tissue remains incompletely understood, particularly regarding stress-adaptive macrophage programs linked to proteostasis imbalance [13,14] and their spatial roles in extracellular matrix remodeling and immune suppression [10,15,16]. This gap likely limits the development of effective macrophage-targeted therapies in HCC [11,12,17]. While spatial transcriptomics (ST) offers new opportunities to study immune processes in intact tissue by preserving spatial context [18], the resolution of ST platforms varies, and widely used sequencing-based methods such as 10x Visium platform (10x Genomics, Pleasanton, CA, USA) do not provide single-cell resolution. Conversely, scRNA-seq captures cellular states but fails to provide spatial information [9,18,19]. Integrating these complementary modalities is therefore critical for linking cell identity, functional state, and spatial localization, and for advancing spatially informed therapeutic strategies in HCC.

In this study, we integrated single-cell and spatial transcriptomic analyses to characterize TAM heterogeneity in HCC. We identified five TAM subclusters, and focused on a less well-characterized BAG3^+^ TAMs with a coordinated transcriptional program involving stress adaptation, proteostasis, and matrix remodeling. Regulatory and intercellular communication analyses further suggested its association with an immunosuppressive microenvironment in HCC. Finally, we derived a *BAG3*-associated risk score that served as an independent prognostic factor in HCC and was associated with immune contexture and drug-response patterns. Together, our study provides an integrated single-cell and spatial atlas of TAM states in HCC and further characterizes a *BAG3*-associated TAM program in the HCC context, providing a basis for future mechanistic and biomarker studies.

## 2. Materials and Methods

### 2.1. scRNA-Seq Datasets and Analysis

Two publicly available scRNA-seq datasets, comprising 41 samples from 20 patients in total, were used in this study for discovery and validation analyses. The discovery dataset, reported by Lu et al. [7], comprised 21 samples from 10 patients with HCC across multiple tissue sites, including primary tumor, adjacent non-tumor liver, lymph-node metastasis, and portal vein tumor thrombus (PVTT), and was used for cellular landscape characterization and TAM heterogeneity analysis. The second dataset, reported by Giraud et al. [20], comprised 20 paired tumor-adjacent samples from 10 patients and was used for validation. Raw sequencing data were processed using Cell Ranger (10x Genomics, Pleasanton, CA, USA) and analyzed in R (v4.4.0) with Seurat (v5.1.0) [21]. Myeloid-lineage cells were further subsetted, and an integrated validation dataset for paired tumor-adjacent analyses was generated. Detailed procedures are provided in Supplementary Note S1.

### 2.2. Inference of Copy-Number Variations and Classification of Malignant Cells

Large-scale copy-number variations (CNVs) were inferred from scRNA-seq data using inferCNV (v1.22.0) [22,23] to distinguish malignant from non-malignant hepatocytes using immune and stromal cells as references. Malignancy was determined using a reference-anchored CNV amplitude score to control false positives among non-tumor cell types. Detailed inferCNV procedures are provided in Supplementary Note S2.

### 2.3. Differential Expression, Functional Enrichment, and Metabolic Activity Analyses

Differentially expressed genes (DEGs) were identified using Seurat’s FindAllMarkers or FindMarkers functions, and genes with an absolute log_2_ fold change (|log_2_FC|) ≥ 1 and a false discovery rate (FDR) < 0.05 were considered significant. For the comparison between malignant and non-malignant hepatocytes, differential expression was performed using a descriptive Wilcoxon rank-sum test without explicit adjustment for viral or etiologic covariates. Functional enrichment analyses, including Gene Ontology Biological Process (GO-BP) and Kyoto Encyclopedia of Genes and Genomes (KEGG) analyses, were performed using clusterProfiler (v4.12.6) [24], with human gene annotation from org.Hs.eg.db (v3.19.1). Metabolic activity of five TAM subsets was quantified using scMetabolism (v0.2.1) [19].

### 2.4. TAM Context Preference and Pseudotime Trajectory Inference

The preference of TAM subsets for HCC etiologies and tissue sites was quantified using the STARTRAC-dist index, as described by Zhang et al. [25]. Pseudotime analysis was performed in hepatitis B virus (HBV)-derived myeloid cells using Monocle2 (v2.32.0) [26], with VCAN^+^ monocytes designated as the root state. MMP9^+^ TAMs were excluded because of their underrepresentation in HBV samples. Pseudotime-associated genes were grouped into temporal modules using ClusterGVis (v0.1.4) and further annotated by GO-BP enrichment analysis. For sensitivity analysis, pseudotime inference was additionally performed in non-HBV-derived TAMs, including HCV and NBNC samples, using the same Monocle2-based workflow. Detailed procedures are provided in Supplementary Note S3.

### 2.5. Regulatory and Cell–Cell Communication Analyses

Gene regulatory activity in TAMs was quantified using the Single-Cell Regulatory Network Inference and Clustering (SCENIC) framework proposed by Aibar et al. [27], implemented in version 1.3.1. Cell–cell communication was inferred using CellChat (v2.2.0) [28] based on Seurat-normalized expression values, curated cell-type annotations, and the built-in human ligand-receptor database. Downstream transcriptional coupling was further assessed using cellcall (v1.0.7) [29], which links receptor-side signaling to transcription factor (TF) activity. Additional details are provided in Supplementary Note S3.

### 2.6. Spatial Transcriptomics Analysis and Multi-Algorithm Spatial Scoring

Spatial transcriptomics data were retrieved from the NCBI Gene Expression Omnibus (GEO; GSE238264), as previously reported by Zhang et al. [30], and included four immune-enriched HCC sections derived from patients treated with neoadjuvant cabozantinib plus nivolumab, comprising three responder sections (ST01-R, ST02-R, and ST03-R) and one non-responder section (ST04-NR). For gene-set activity estimation, AUCell (v1.26.0) [27], UCell (v2.9.0) [31], and Seurat AddModuleScore [21] were applied, and their outputs were integrated by robust z-score normalization followed by equal-weight averaging to generate a composite metaZ score. Cells or spatial spots were stratified into high- and low-score groups using percentile-based cutoffs, and between-group differences were assessed using two-sided Wilcoxon tests with FDR correction. Additional details are provided in Supplementary Note S4.

### 2.7. Bulk RNA-Seq Processing, Prognostic Modeling, and Downstream Analyses

Bulk RNA sequencing (RNA-seq) expression profiles and clinical annotations for The Cancer Genome Atlas Liver Hepatocellular Carcinoma cohort (TCGA-LIHC) [32] were downloaded via the Genomic Data Commons portal, and the external International Cancer Genome Consortium Liver Cancer–Riken, Japan cohort (ICGC-LIRI-JP) [33] was obtained from the ICGC data portal. Analyses were restricted to primary tumors with complete survival information, and expression values quantified as transcripts per million (TPM) were transformed as ${log}_{2}(TPM+1)$. To construct a *BAG3*-associated prognostic signature, candidate genes were first screened using univariate Cox proportional hazards models implemented with survival (v3.5-8), and an elastic-net Cox model implemented with glmnet (v4.1-8) was then applied to define a 10-gene signature. Four survival models were fitted, including a multivariable Cox model using survival (v3.5-8), a random survival forest (RSF) model constructed with randomForestSRC (v3.4.1), an Extreme Gradient Boosting (XGBoost) survival model fitted with xgboost (v1.7.11.1), and a survival support vector machine (SVM) model using survivalsvm (v0.0.6). Patients were stratified by the median risk score (RS), and model performance was evaluated using Kaplan–Meier analysis with survival (v3.5-8) and survminer (v0.5.0), Harrell’s concordance index (C-index), and time-dependent receiver operating characteristic (ROC) curves with the corresponding areas under the curve (AUCs) using timeROC (v0.4). Immune infiltration was estimated using Cell-type Identification By Estimating Relative Subsets Of RNA Transcripts (CIBERSORT) [34], immune checkpoint activity was quantified using single-sample gene set enrichment analysis (ssGSEA) [35] implemented in GSVA (v1.52.3), drug sensitivity was predicted using oncoPredict (v1.2) [36], and pathway activity was quantified using Reactome and Hallmark gene sets obtained through msigdbr (v25.1.1). All the above-mentioned R packages are conducted on R (v4.4.0). Additional details are provided in Supplementary Note S4.

## 3. Results

### 3.1. Single-Cell and Spatial Transcriptomic Profiling of Hepatocellular Carcinoma

**Figure 1:**
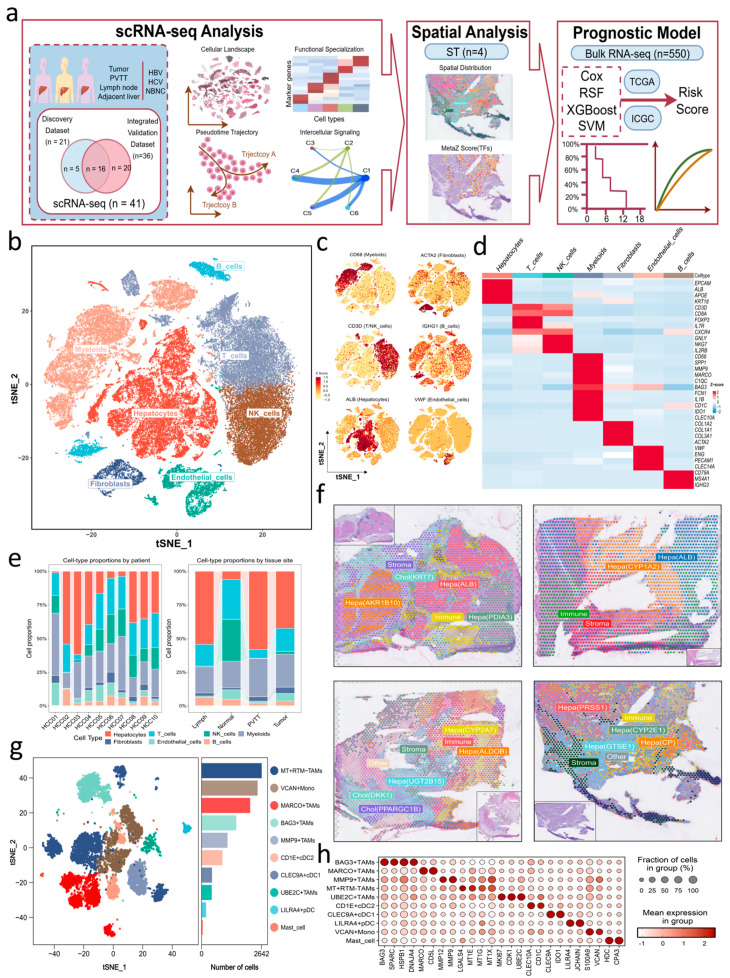
Integrated single-cell and spatial transcriptomic landscape of HCC and myeloid heterogeneity. (**a**) Schematic overview of study design and data modalities. (**b**) The t-SNE plot showing the distribution of major cell types. (**c**) Feature plots of representative marker genes of major cell types on the t-SNE. A more complete marker-gene panel is provided in Supplementary Figure S1c. (**d**) Heatmap of top marker genes of the annotated cell types. (**e**) Major cell-type composition stratified by patient and tissue sites, based on major cell-type annotation. Proportions are shown among high-quality cells retained after quality control. (**f**) Spatial maps with spot-level annotations, including hepatocyte-related domains (e.g., *ALB*, *CYP1A2* and *AKR1B10*), a cholangiocyte-like compartment (e.g., *KRT7* and *DKK1*), and surrounding immune/stromal regions. Insets show the corresponding H&E images. (**g**) t-SNE of the myeloid compartment showing myeloid subsets. (**h**) Bubble plot of marker gene expression across myeloid subsets.

To establish a comprehensive single-cell transcriptomic landscape of HCC, we analyzed a discovery scRNA-seq dataset comprising 21 tissue samples from 10 patients, including primary tumors, adjacent non-tumor liver, PVTT, and lymph-node metastases, and representing major etiologic groups such as HBV, hepatitis C virus (HCV), and non-viral (NBNC) cases (Figure 1a; Supplementary Table S1). After quality control, 70,749 high-quality cells and 25,479 genes were retained for downstream analysis (Supplementary Figure S1a,b). These cells resolved into seven major cell types based on canonical marker expression, including hepatocytes, fibroblasts, endothelial cells, myeloid cells, T cells, natural killer (NK) cells, and B cells, and were visualized on a t-distributed stochastic neighbor embedding (t-SNE) projection (Figure 1b). Canonical marker genes showed consistent lineage-specific patterns which supported the assigned identities (Figure 1c,d; Supplementary Figure S1c). Among recovered high-quality cells, hepatocytes showed marked inter-patient variability and were relatively underrepresented in adjacent non-tumor samples while being more abundant in tumor-related sites, including primary tumors, PVTT, and lymph-node metastases (Figure 1e). In contrast, T and NK cells were more abundant in adjacent non-tumor liver but reduced in tumor-related sites, whereas myeloid cells were increased in primary tumors and PVTT. Together, these patterns suggest an immunosuppressive microenvironment across tumor sites, consistent with the role of myeloid lineages in promoting T cell dysfunction and tumor progression [37,38].

To assess reproducibility, we next analyzed an integrated validation dataset comprising 36 tissue samples from 18 patients with HCC (Supplementary Table S1). After quality control, 139,081 high-quality cells and 30,654 genes were retained (Supplementary Figure S2). The major cell lineages and marker-defined populations were consistently recapitulated, supporting the robustness of the overall cellular landscape. To complement the single-cell atlas with spatial context, we further analyzed four immune-enriched HCC sections from an independent spatial transcriptomics dataset (Supplementary Table S2). Major spatial domains were annotated using marker frameworks consistent with the scRNA-seq analysis, and representative markers are shown for selected hepatocyte-related domains and the cholangiocyte-like compartment (Figure 1f; Supplementary Note S4). Given the myeloid-enriched immune niche surrounding tumor hepatocytes identified by single-cell and spatial analyses, we next characterized the myeloid compartment to dissect its heterogeneity and immune regulatory functions.

### 3.2. Myeloid Cell Heterogeneity in HCC

**Figure 2:**
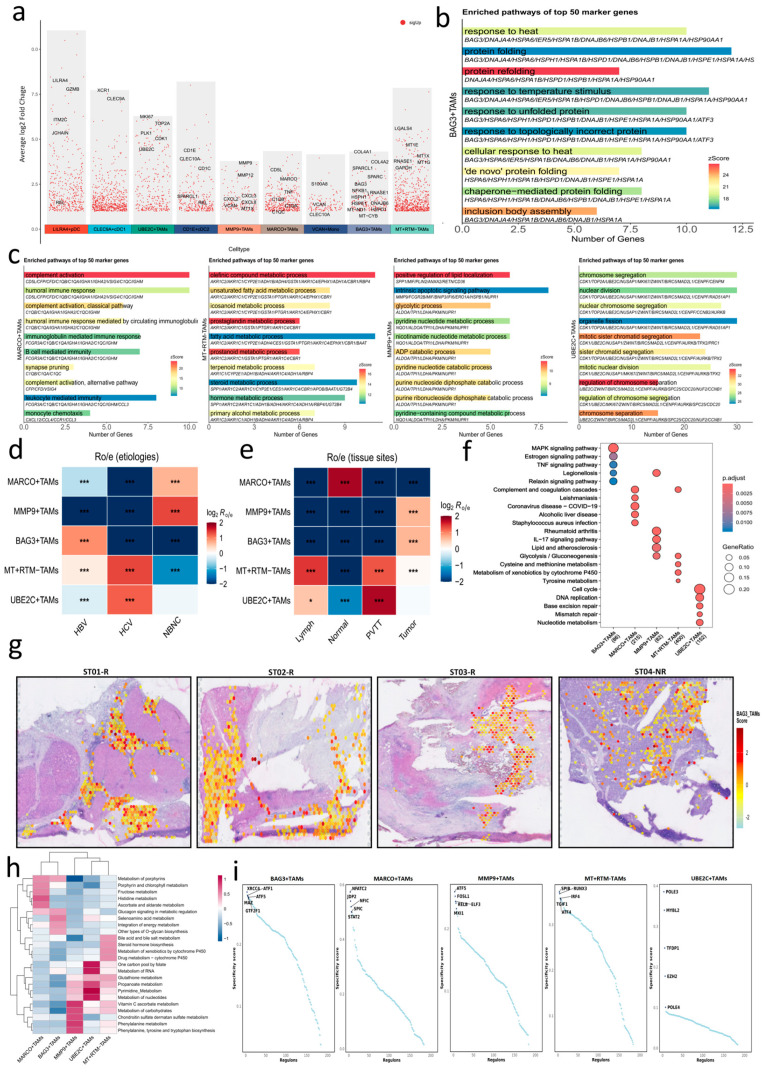
Heterogeneity of distinct TAMs in HCC. (**a**) Distribution of significantly upregulated marker genes across myeloid subsets from one-versus-rest differential expression analysis. Red dots represent individual genes positioned by average log2 fold change; labeled genes indicate representative markers. Gray background columns visually group each subset. (**b**,**c**) GO-BP enrichment analysis of pathways enriched among marker genes upregulated in BAG3^+^ TAMs (**b**) and in MARCO^+^, MT^+^ RTM−, MMP9^+^, and UBE2C^+^ TAMs (**c**). The top GO-BP terms are provided in Supplementary Data S2. (**d**,**e**) Heatmaps showing $log_{2}Ro/e$ of TAM subset enrichment across etiologies (**d**) and tissue sites (**e**). Red, blue, and white indicate enrichment $log_{2}Ro/e>0$, depletion $log_{2}Ro/e<0$, and no clear preference $log_{2}Ro/e\approx 0$, respectively. Asterisks indicate significance after Benjamini–Hochberg correction of chi-squared test *p* values (* FDR<0.05, *** FDR<0.001). (**f**) KEGG pathway analysis highlighting the top five differentially enriched pathways across TAM subsets. Full KEGG results are provided in Supplementary Data S2. (**g**) Spatial mapping of the BAG3^+^ TAM-related metaZ scores in H&E-stained sections, quantified as integrated metaZ scores of the top 50 marker genes, showing preferential localization of this *BAG3*-centered program within immune regions. (**h**) Metabolic pathway analysis highlighting the top five differentially enriched metabolic pathways across TAM subsets, based on the KEGG and Reactome databases. (**i**) SCENIC analysis showing subset-specific regulon activity across TAM subsets.

To delineate macrophage heterogeneity in HCC, we reclustered myeloid-lineage cells from the major cell atlas (Figure 1b), which identified five TAM subsets, including MARCO^+^, MT^+^ resident tissue macrophage-like (MT^+^ RTM−), MMP9^+^, UBE2C^+^, and BAG3^+^ TAMs, as well as three dendritic cell (DC) subsets (CLEC9A^+^ cDC1, CD1E^+^ cDC2, and LILRA4^+^ pDC), monocytes, and mast cells (Figure 1g,h and Figure 2a; Supplementary Figure S3a,b). Full differential expression results for the five TAM subsets are provided in Supplementary Data S1. For comparison with previously reported macrophage states [10,39], representative known TAM subtype markers, including *SPP1*, *SEPP1*, *TREM2*, and *APOE*, are shown in Supplementary Figure S3b,c. GO-BP enrichment analysis highlighted a stress-adaptive, proteostasis-related, and remodeling-associated program in BAG3^+^ TAMs (Figure 2b; Supplementary Data S2), alongside distinct functional enrichments across the other four TAM subsets (Figure 2c; Supplementary Data S2). MARCO^+^ TAMs showed elevated Kupffer-cell markers [10] (*CD5L*, *MARCO*, and *C1QA/B/C*) (Figure 1h), and were enriched for complement activation and leukocyte-mediated immunity, consistent with a homeostatic resident-like program [40]. In contrast, MT^+^ RTM− TAMs co-expressed metallothioneins (*MT1X/G/E*) and residency markers [41] (*TREM2*) (Figure 1h), with top enriched GO-BP terms involving mitochondrial, detoxification, toxic-response, and lipid/steroid-related metabolic processes, consistent with metallothionein-high macrophage states [42]. MMP9^+^ TAMs displayed a pro-inflammatory profile with elevated *MMP9/12* and chemokines *CXCL2/3/8* (Figure 1h), reflecting stromal-remodeling TAM programs reported across solid tumors, including gastric cancer [43], and showed enrichment of glycolytic, intrinsic apoptotic, pyridine/nicotinamide nucleotide metabolic, secretion-related, and wound-healing-associated processes, consistent with an inflammatory matrix remodeling program. UBE2C^+^ TAMs exhibited a proliferative program, marked by *UBE2C* together with cell-cycle regulators [10] (*CDK1*, *MKI67*, *TOP2A*, and *PLK1*) (Figure 1h), with enrichment for mitotic pathways including chromosome segregation, nuclear division, and organelle fission (Figure 2c), consistent with cycling macrophage subsets across multiple cancers [44,45]. The BAG3^+^ TAMs were characterized by the expression of stress-adaptive genes (*BAG3*, *HSPH1*, *HSPD1*, *HSPE1*, and *DNAJB6*) and matrix-regulatory factors (*SPARC*, *SPARCL1*, *COL4A1*, and *COL4A2*) (Figure 1h). Representative GO-BP terms highlighted heat-response and chaperone-related pathways (Figure 2b), while the top enriched pathways further supported cytokine/TNF-related and vasculature-associated features (Supplementary Data S2). While their chaperone-mediated protein-folding signature overlapped with BAG3^+^ TAM programs reported in colorectal cancers [46] and gastric cancers [47], a *BAG3*-centered TAM program has not been well characterized in HCC. This subset further showed increased mitochondrial-stress modules (*MT-CYB*, *MT-ND3*, and *MT-CO3*) and enrichment for TNF-α/NF-κB signaling involving NFKB1 and REL (Figure 2b), consistent with adaptation to metabolic and oxidative stress within the HCC microenvironment. In parallel with TAM specialization, the myeloid compartment also contained CLEC9A^+^ cDC1, CD1E^+^ cDC2, and LILRA4^+^ pDC subsets, corresponding to antigen cross-presentation, CD4^+^ T-cell immunity, and type I interferon-mediated antiviral and antitumor responses [48,49]. VCAN^+^ monocytes showed a chemokine-rich phenotype [39], whereas sparse mast cells may contribute to protease-dependent immunomodulation and stromal remodeling [50] (Figure 1g,h).

### 3.3. Functional Specialization of TAM Subsets

We first characterized TAM distributions across etiologic groups and tissue sites in the discovery dataset. STARTRAC-dist [25] analysis showed that five TAM subsets were not uniformly distributed across HBV-, HCV-, and NBNC-HCC (Figure 2d). In particular, MMP9^+^ TAMs were predominantly enriched in NBNC-HCC, consistent with their inflammatory and matrix-remodeling features [43,51], whereas MT^+^ RTM− and UBE2C^+^ TAMs were detected across all etiologies but were relatively more abundant in HCV-related HCC. BAG3^+^ TAMs appeared increased in HBV-associated tumors, although this trend requires validation in independent HBV-enriched cohorts. Across tissue sites, Kupffer cell-like (MARCO^+^) TAMs predominated in adjacent non-tumor liver but were scarce in PVTT and lymph nodes, consistent with their sinusoidal residency [52]. By contrast, MMP9^+^ and BAG3^+^ TAMs were preferentially enriched in primary tumors, whereas MT^+^ RTM− and UBE2C^+^ TAMs showed relative enrichment in PVTT (Figure 2e). To assess the reproducibility of the *BAG3*-associated program, we analyzed the integrated validation scRNA-seq dataset comprising paired tumor-adjacent samples (see validation dataset assembly and preprocessing in Supplementary Figure S2; myeloid cell subcluster analysis in Supplementary Figure S4) and performed myeloid-specific re-clustering. This analysis identified a distinct *BAG3*-enriched cluster (C7) with stress-chaperone and monocyte/macrophage features (Supplementary Figure S4a–d). C7 was detectable across etiologies and in both tumor and matched adjacent tissues, although its abundance varied substantially between individuals (Supplementary Figure S4b,e). Functional analysis showed enrichment of protein homeostasis, antigen processing and lysosomal activity, MAPK signaling, and lipid metabolism (Supplementary Figure S4f,g). These features recapitulated the GO-BP and KEGG patterns observed for BAG3^+^ TAMs in the discovery dataset (Figure 2b,f), supporting cross-dataset reproducibility of the core stress-response program. In contrast, extracellular matrix-related signals showed lower stability in the integrated analysis and warrant verification in expanded cohorts (Supplementary Figure S4h,i). Spatial transcriptomic mapping showed preferential localization of this *BAG3*-centered program within immune regions. This pattern was consistent with the myeloid and immune associated context of the *BAG3* program identified in the single-cell analysis, thereby providing supportive spatial context for the single-cell results in a small treatment-exposed cohort (Figure 2g).

KEGG enrichment of subset markers (Figure 2f; Supplementary Data S2) together with scMetabolism [19] quantification (Figure 2h) indicated a five-part metabolic division of labor across TAM subsets. MARCO^+^ TAMs occupied a homeostatic end of the spectrum, with low global metabolic flux and complement/coagulation features [10,53]. BAG3^+^ and MMP9^+^ TAMs both showed enrichment of inflammatory pathways, including TNF and IL-17 signaling, but with different dominant functional orientations. BAG3^+^ TAMs were linked more closely to MAPK-associated stress responses and stress-chaperone/proteostasis-related immunoregulation, whereas MMP9^+^ TAMs were characterized more by chemokine signaling, carbohydrate metabolism, and matrix-/wound-healing-associated remodeling programs. UBE2C^+^ TAMs showed proliferative programs supported by one-carbon and nucleotide metabolism [10]. MT^+^ RTM− TAMs were enriched for liver-centric detoxification, including lipid handling, bile-acid metabolism, and cytochrome P450-mediated xenobiotic clearance [54]. Together, these results define multi-axis TAM specialization spanning homeostasis, stress/proteostasis, matrix-inflammation, proliferation, and detoxification.

SCENIC analysis further identified subset-specific master regulons (Figure 2i). Stress-responsive transcription factors [55,56,57] (ATF1, ATF4, and ATF5) were enriched in BAG3^+^, MMP9^+^, and MT^+^ RTM− TAM subsets (Figure 2i; Supplementary Data S3), linking these states to adaptation against hypoxia, oxidative stress, and metabolic stress. Regulons linked to immune homeostasis [58,59] (STAT2, SPIC, and NFATC2) and cell cycle regulation (MYBL2, TFDP1, and EZH2) were preferentially enriched in MARCO^+^ TAMs and UBE2C^+^ TAMs, respectively (Figure 2i; Supplementary Data S3). In addition, MAZ, FOSL1, RELB, and MXI1 were enriched in BAG3^+^ and MMP9^+^ TAMs, suggesting roles in pro-inflammatory signaling and stress adaptation which may contribute to an immunosuppressive milieu [60,61].

### 3.4. Malignant Hepatocytes Show a Shift from Metabolism to Proliferation

**Figure 3:**
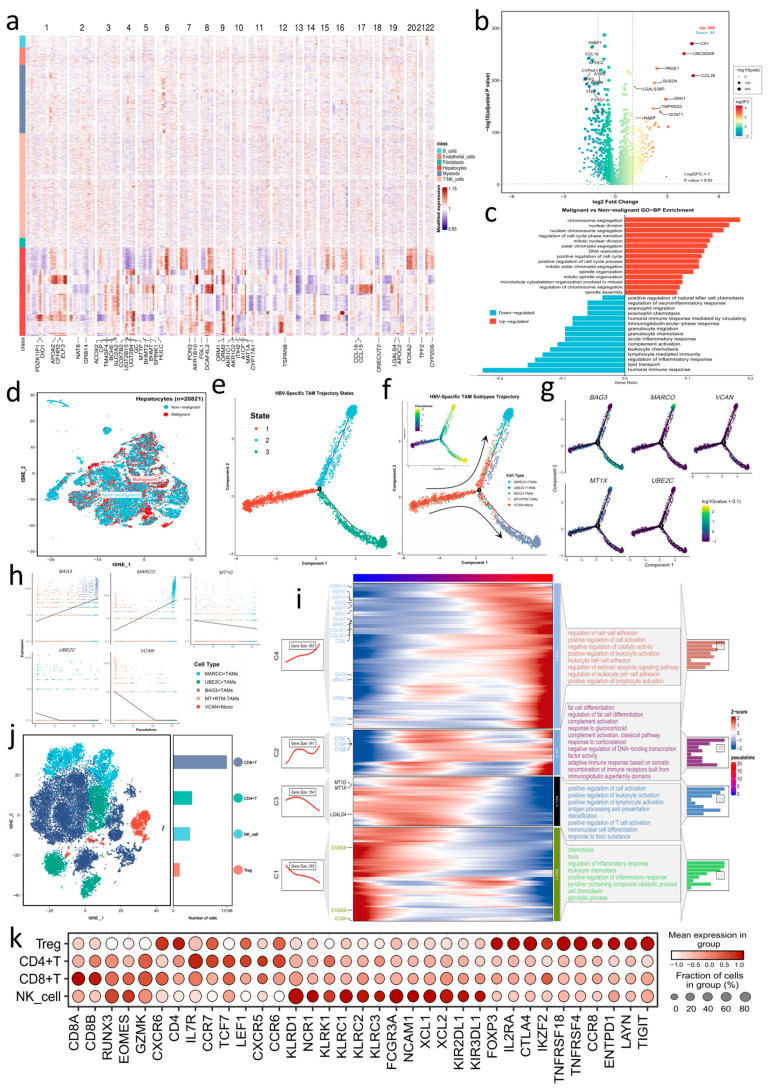
CNV-based identification of malignant hepatocytes and TAMs trajectories with the T/NK immune context in HCC. (**a**) inferCNV heatmap classifying hepatocytes as malignant or non-malignant using non-parenchymal cells as references; the feature strip indicates the most variable genes used. (**b**) Volcano plot of DEGs between malignant and non-malignant hepatocytes (Wilcoxon test; FDR<0.05;$\;|{log}_{2}FC|\geq 1$); top 10 upregulated and downregulated genes are labeled. (**c**) GO-BP enrichment of genes upregulated or downregulated in malignant hepatocytes. (**d**) t-SNE of hepatocytes colored by malignant status. (**e**,**f**) Pseudotime trajectories of myeloid cells colored by inferred states (**e**) and TAM subsets (**f**), with arrows indicating differentiation from VCAN^+^ monocytes. (**g**,**h**) Branch-wise expression patterns (**g**) and pseudotime trends (**h**) of selected genes (*BAG3*, *MARCO*, *MT1X*, *UBE2C* and *VCAN*). (**i**) Heatmap of pseudotime-associated genes grouped into four modules (C1–C4) with representative GO terms. (**j**) t-SNE of T/NK cells colored by subsets (CD8^+^ T, CD4^+^ T, Treg and NK cells), with a sidebar indicating subset sizes; a subset of initial annotated NK cells was reassigned to T-cell lineages based on transcriptomic profiles. (**k**) Dot plot of signature and checkpoint genes across T/NK subsets.

We delineated hepatocyte states within the tumor microenvironment by applying inferCNV [22,23] (as detailed in Section 2), using multiple non-parenchymal cell types as reference populations. Hepatocytes were classified as malignant or non-malignant based on widespread copy number alterations, which were summarized by CNV scores (Figure 3a; Supplementary Data S4). Using a Wilcoxon test with thresholds of FDR<0.05 and $\left|{log}_{2}FC\right|\geq 1$ (Figure 3b), comparative transcriptomic analysis between the two groups identified 266 upregulated and 95 downregulated genes in malignant hepatocytes, indicating substantial transcriptional reprogramming. Upregulated genes were enriched for cell-cycle, DNA replication, and extracellular matrix-related pathways (e.g., *DKK1*, and *TMPRSS3*), consistent with proliferative and remodeling features commonly observed in cancer [62]. In contrast, the downregulated genes were enriched for metabolic functions (e.g., *FABP1* and *APOC* family), indicating reduced tissue-specific metabolic activity in line with metabolic rewiring observed across cancers and in HCC [63]. GO-BP analysis supported this pattern, showing coordinated activation of proliferation pathways together with suppression of metabolic pathways (Figure 3c). The t-SNE embedding showed malignant hepatocytes distributed across adjacent neighborhoods, indicating gradual deviation from mature states, paralleled by increased CNV burden and transcriptomic reprogramming, consistent with hepatocyte dedifferentiation in liver disease and cancer progression (Figure 3d) [64].

### 3.5. Pseudotime Dynamics of TAMs in a Representative HCC Subset

To examine transcriptional continuity under comparable sampling conditions, we inferred pseudotime using a subset with dense coverage across TAM states (predominantly from HBV samples). The trajectory was rooted in VCAN^+^ monocytes (Figure 3e,f) and bifurcated after a shared transitional region into a resident branch progressing through MT^+^ RTM− states to MARCO^+^ TAMs and a stress-remodeling branch advancing toward BAG3^+^ TAMs. This topology is consistent with myeloid fate bifurcations reported previously [37,65,66], although the trajectory observed here showed distinct branch-specific features. Within this trajectory, UBE2C^+^ TAMs were observed at low frequency and did not form a stable terminal state. Gene dynamics followed the branching structure (Figure 3g,h): *VCAN* declined from the root; *MARCO* and *C1QA/B/C* increased along the resident branch. By contrast, the branch leading toward BAG3^+^ TAMs, hereafter referred to as the stress-remodeling branch, showed a late increase in *BAG3*, co-expressed HSP chaperones (*HSPH1*, *HSPD1*, *HSPE1*, and *DNAJB1*), together with ECM/adhesion genes (*SPARC*, *COL4A1/2*, *PLVAP*, and *IGFBP7*). Meanwhile, MT family genes (e.g., *MT1X* and *MT1G*) decreased over pseudotime, and *UBE2C* attenuated early. To further characterize the temporal progression, pseudotime-associated genes were grouped into four temporal modules (Figure 3i), capturing a continuum from chemotaxis/inflammatory recruitment and glycolysis (e.g., *VCAN* and *S100A8/A9*), through antigen processing with metallothionein buffering (e.g., *MT1G/MT1X*), to complement/lipid-handling tolerance states (e.g., *C1QA/B/C*), and finally to chaperone-mediated stress responses coupled with ECM/adhesion pathways (BAG3-HSP-ECM module), consistent with increasing immune–stromal coupling [67]. Notably, C1Q genes peaked at mid-to-late stages along the resident path, whereas *CD5L* and the BAG3-HSP-ECM module accumulated later; *MARCO* and *C5AR1* increased in a transitional window between clearance/tolerance and stress remodeling. As an exploratory sensitivity analysis, we also inferred pseudotime in non-HBV-derived TAMs, including HCV and NBNC samples, where MMP9^+^ TAMs were more represented. This analysis showed that MMP9^+^ TAMs were mainly located outside the MARCO^+^-enriched branch and partially overlapped with other TAM subsets, without forming a clearly separated terminal branch (Supplementary Figure S5).

### 3.6. BAG3^+^ TAMs Are Associated with Stress-Related Immunoregulatory Signaling

**Figure 4:**
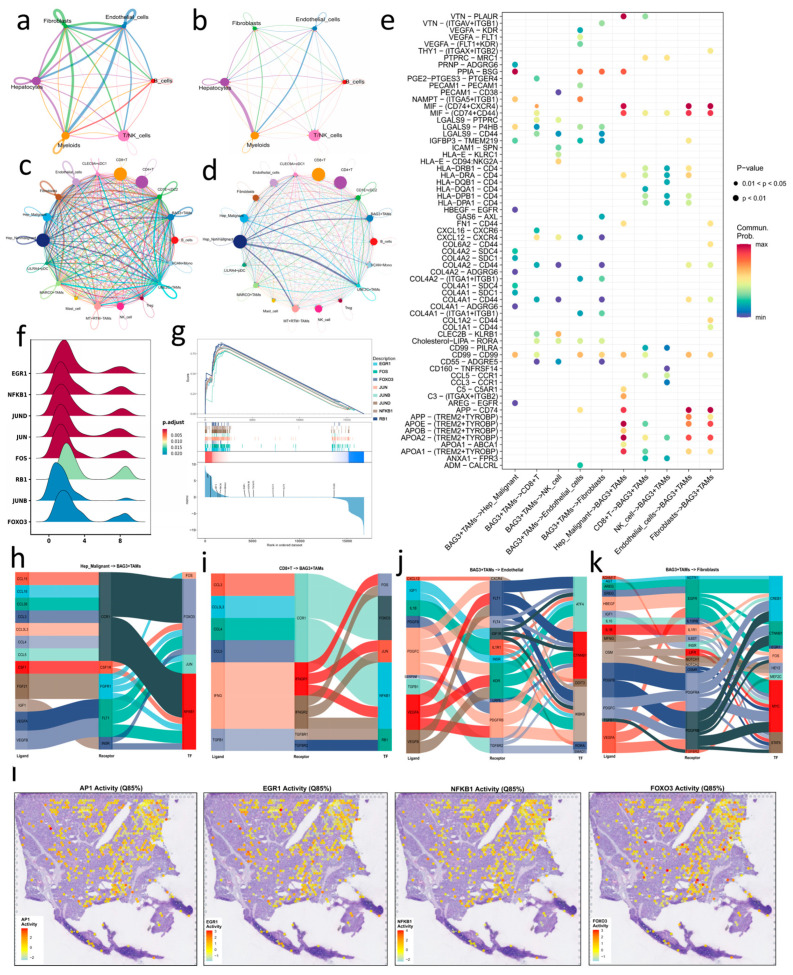
BAG3^+^ TAMs show enriched signaling interactions across multicellular circuits. (**a**,**b**) CellChat networks showing (**a**) the number and (**b**) the interaction strength of significant ligand–receptor pairs among major cell types; hepatocytes are shown here as a combined lineage-level compartment. (**c**,**d**) Outgoing (**c**) and incoming (**d**) communication patterns at subcluster resolution, showing inferred outgoing and incoming communication patterns involving BAG3^+^ TAMs across malignant and non-malignant hepatocytes, multiple TAM subsets, and other finer stromal and immune populations. (**e**) Bubble plot of representative ligand–receptor interactions involving BAG3^+^ TAMs, including interactions with malignant hepatocytes; corresponding interactions with non-malignant hepatocytes are shown in Supplementary Figure S6f. (**f**,**g**) TF activity in BAG3^+^ TAMs, shown by (**f**) ridge plots of key TFs scores (EGR1, AP-1, NFKB1, FOXO3) and (**g**) a representative GSEA plot for a TF regulon. (**h**–**k**) CellCall Sankey diagrams mapping representative ligand–receptor–TF circuits, including signaling from malignant hepatocytes to BAG3^+^ TAMs (**h**), from CD8^+^ T cells to BAG3^+^ TAMs (**i**), from BAG3^+^ TAMs to endothelial cells (**j**), and from BAG3^+^ TAMs to fibroblasts (**k**). (**l**) Representative spatial activity maps of AP-1, EGR1, NFKB1, and FOXO3 in ST04-NR are shown to visualize the spatial patterns; complete four-section statistics are provided in Supplementary Table S3.

To investigate the immunoregulatory features and cellular interactome associated with BAG3^+^ TAMs, we first performed high-resolution re-clustering of T and NK cells, which identified CD8^+^ T cells, CD4^+^ T cells, regulatory T (Tregs), and NK cells (Figure 3j), with marker gene profiles that matched those established in multimodal PBMC reference atlases [68] (Figure 3k). CellChat [28] analysis at the major cell-type level showed extensive communication between myeloid cells and hepatocytes, endothelial cells, and fibroblasts, with the myeloid–hepatocyte axis among the strongest lineage-level interactions (Figure 4a,b). To further resolve this communication landscape, we next examined the network at subcluster resolution, in which malignant and non-malignant hepatocytes, multiple TAM subsets, and other finer stromal and immune populations were analyzed separately. At this level, BAG3^+^ TAMs displayed particularly high incoming and outgoing communication across parenchymal, stromal, and immune compartments, suggesting a highly connected position in the inferred HCC communication network (Figure 4c–e; Supplementary Data S5; Supplementary Note S5). Because BAG3^+^ TAMs were relatively sparse in non-HBV tumors, the detailed BAG3^+^ TAM-centered CellChat analyses below focus primarily on HBV-derived samples, with corresponding non-HBV results shown in Supplementary Figures S6a–e and S7. Incoming signals to BAG3^+^ TAMs included apolipoprotein-related cues engaging the TREM2–TYROBP complex, consistent with lipid handling and immunomodulatory signaling [12], as well as MIF–CD74-related signaling, linked to inflammatory survival programs [60,69,70], and PPIA–BSG, which has been associated with extracellular matrix remodeling and vascular permeability [71,72]. Outgoing signaling from BAG3^+^ TAMs was enriched for VEGFA–KDR/FLT1 and integrin-/collagen-associated interactions toward endothelial and fibroblast compartments, consistent with proangiogenic and stromal-remodeling functions, whereas communication toward lymphocytes prominently involved CXCL12–CXCR4 and HLA-E–CD94/NKG2A, suggesting roles in lymphocyte recruitment, retention, and effector regulation [70,71,72]. Notably, MIF–CD74 and HLA-II–CD4 axes also recurred across multiple immune sender–receiver configurations, indicating that these pathways are broadly used within the local immune compartment (Supplementary Figure S6f). Across malignant and non-malignant hepatocyte states, the major BAG3^+^ TAM-centered interaction themes were largely shared. The non-malignant compartment nevertheless showed a somewhat broader repertoire, including additional CXCL12–CXCR4, APOA1–ABCA1, GDF15–TGFBR2, and ANXA1–FPR3 interactions, whereas malignant hepatocytes uniquely displayed ICAM1 − (ITGAX + ITGB2) (Figure 4e; Supplementary Figure S6f; Supplementary Data S5). This pattern may reflect broader homeostatic and paracrine signaling in non-malignant hepatocytes and a more reprogrammed communication state in malignant hepatocytes. These inferred patterns are consistent with an association between BAG3^+^ TAMs, stress-related microenvironmental cues, and stromal or immune niche remodeling.

CellCall [29] analysis further linked these ligand–receptor interactions to downstream transcriptional responses (see Supplementary Note S5 for additional details). BAG3^+^ TAMs showed increased activity of target genes associated with EGR1, NFKB1, and AP-1 (JUN/FOS), together with additional enrichment of FOXO3 and JUNB, consistent with coordinated stress and inflammatory responses [73] (Figure 4f,g). Sankey-based visualization further linked BAG3^+^ TAM signaling to transcriptional circuits centered on NF-κB, AP-1, and FOXO3 (Figure 4h–k; Supplementary Data S6). As receivers, BAG3^+^ TAMs were associated with chemotactic, survival, and inflammatory cues through CCL3/4/5–CCR1, CSF1–CSF1R, IFNG–IFNGR1/2, and TGFβ–TGFBR1/2; as senders, they showed potential proangiogenic, matrix-remodeling, and immunoregulatory signals via VEGFA–KDR/FLT1, TGFβ–TGFBR1/2, MIF–CD74-related signaling, CXCL12–CXCR4, and HLA-E–CD94/NKG2A. Notably, malignant and non-malignant hepatocytes shared a chemokine input centered on CCR1 in BAG3^+^ TAMs but differed in selected axes associated with hepatocyte state. CCL26–CCR1 was observed in malignant hepatocyte circuits toward BAG3^+^ TAMs (Figure 4h), suggesting inflammatory cues related to the tumor state, whereas EDN1–EDNRB was observed in non-malignant hepatocyte circuits toward BAG3^+^ TAMs (Supplementary Figure S6g), consistent with tissue stress or remodeling cues from the tumor-bearing liver. Together, these findings suggest BAG3^+^ TAMs are associated with a stress-adaptive and immunoregulatory signaling program linked to vascular remodeling, matrix reorganization, and immune regulation, particularly in the HBV-derived analysis subset.

### 3.7. Spatial Mapping of TF Activity Profiles in BAG3^+^ TAMs

**Figure 5:**
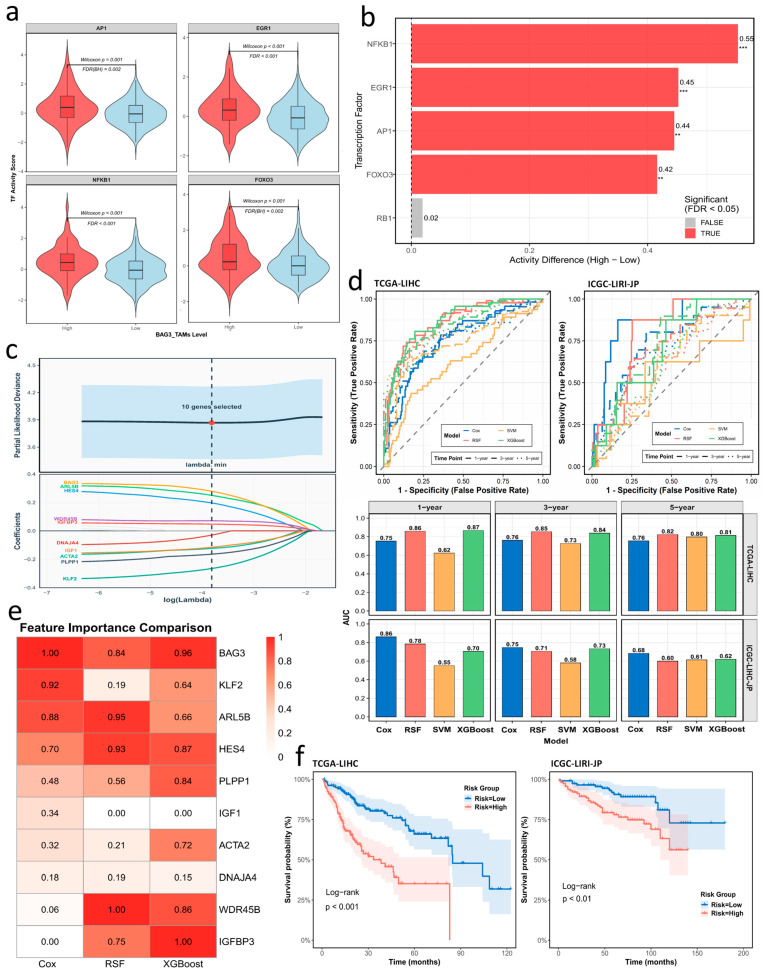
Prognostic evaluation of BAG3^+^ TAM-related TF activity and risk model. (**a**) Representative ST04-NR violin plots of TF activity (AP-1, EGR1, NFKB1, and FOXO3) between BAG3^+^ TAM-high and -low immune regions. (**b**) Representative ST04-NR horizontal bar plot showing metaZ differences for the indicated TFs. BAG3^+^ TAM-high regions were defined as the top 15% spots, and BAG3^+^ TAM-low regions as the remaining 85% spots. Complete statistics across all four ST sections are provided in Supplementary Table S3. ** FDR < 0.01, *** FDR < 0.001. (**c**) Elastic-net Cox cross-validation curve of partial-likelihood deviance across log(λ); the dashed line and red point indicate lambda.min, and the value of the minimum cross-validated deviance used to select the final 10-gene signature, respectively. Coefficient paths below show shrinkage, with gene labels shown on the left. (**d**) Time-dependent receiver operating characteristic curves comparing 1-, 3-, and 5-year overall survival prediction across four models (multivariable Cox, RSF, XGBoost, and SVM) in TCGA-LIHC (training data) and the ICGC-LIRI-JP cohort (validation data); AUCs are shown in the faceted bar-plots below. (**e**) Heatmap of normalized feature importance of the *BAG3*-associated signature (10 genes) across the multivariable Cox, RSF, and XGBoost models, quantified using the model-specific feature importance measures. (**f**) Kaplan–Meier curves for high- and low-risk groups stratified by the risk score in TCGA-LIHC and ICGC-LIRI-JP (shaded areas: 95% confidence intervals).

In spatial transcriptomic analyses, immune regions were stratified by the BAG3^+^ TAM metaZ score, and inferred TF activity was compared between BAG3^+^ TAM-high and -low groups across all four ST sections, with complete statistics provided in Supplementary Table S3 and Supplementary Note S6. Across the four sections, AP-1 and EGR1 showed the most consistent increases in BAG3^+^ TAM-high regions, with patterns generally consistent across upper-quantile cutoffs, whereas NFKB1 and FOXO3 showed supportive but more variable increases, and RB1 showed no consistent association. In ST01-R and ST03-R, both AP-1 and EGR1 remained significant across the 75th, 80th, and 85th percentile cutoffs, whereas in ST02-R, EGR1 remained consistently significant and AP-1 reached significance under selected cutoffs. Representative spatial activity maps from ST04-NR are shown in Figure 4l to visualize these patterns. In ST04-NR under the 75th-percentile cutoff, AP-1 and EGR1 were both significantly increased in BAG3^+^ TAM-high regions (mean/median difference = 0.343/0.279, FDR = 4.9 × 10^−3^; and 0.382/0.406, FDR = 1.0 × 10^−4^, respectively; Supplementary Table S3). NFKB1 also remained significant in ST04-NR under the 75th and 85th percentile cutoffs (FDR = 1.39 × 10^−4^ and 8.42 × 10^−5^, respectively). Together with the representative ST04-NR violin plots and bar plot (Figure 5a,b), these spatial metaZ differences support a coordinated *BAG3*-associated stress and inflammatory remodeling axis mainly involving AP-1, EGR1, and NFKB1, with additional contribution from FOXO3. These observations motivated the subsequent prognostic analyses and risk modeling.

### 3.8. BAG3-Associated Prognostic Modeling in HCC

Starting from 50 candidate marker genes of BAG3^+^ TAMs, we first fitted univariable Cox proportional hazards models with overall survival as the endpoint in the TCGA-LIHC cohort. After quality control, 319 patients were retained, and 13 genes showed significant associations with overall survival (*p* < 0.05; Supplementary Table S4). These genes were entered into an elastic-net Cox model (α = 0.5) with 10-fold cross-validation, yielding a 10-gene *BAG3*-associated signature with non-zero coefficients (Figure 5c). Kaplan–Meier analyses confirmed the prognostic relevance of these genes (Supplementary Figure S8a). Based on coefficient directions and expression patterns (Supplementary Figure S8b,c), the signature was further divided into risk-associated genes (*BAG3*, *ARL5B*, *HES4*, *WDR45B*, and *IGFBP3*) and protective genes (*KLF2*, *PLPP1*, *ACTA2*, *IGF1*, and *DNAJA4*).

Using these 10 genes, we trained four survival models in TCGA-LIHC: a multivariable Cox model, RSF, XGBoost, and SVM. Their performance was compared using time-dependent AUCs at 1, 3, and 5 years (Figure 5d). In TCGA-LIHC, XGBoost (AUC = 0.867) and RSF (AUC = 0.859) outperformed the multivariable Cox model (AUC = 0.753) at 1 year, whereas SVM showed the lowest discrimination (AUC = 0.623). However, when evaluated in ICGC-LIRI-JP, the machine-learning models showed reduced discrimination, whereas the multivariable Cox-based RS remained more stable across cohorts. Consistently, C-index identified the multivariable Cox-based RS as the most robust model in both TCGA-LIHC and ICGC-LIRI-JP (0.700 and 0.694, respectively; Supplementary Figure S8d). Given its more stable cross-cohort C-index and explicit coefficient-based interpretability, the multivariable Cox model was selected for final RS construction. Feature-importance analyses further showed that *BAG3*, *ARL5B*, and *HES4* ranked among the top contributors in the multivariable Cox model, RSF, and XGBoost (Figure 5e; Supplementary Figure S8e,f; Supplementary Table S5).

**Figure 6:**
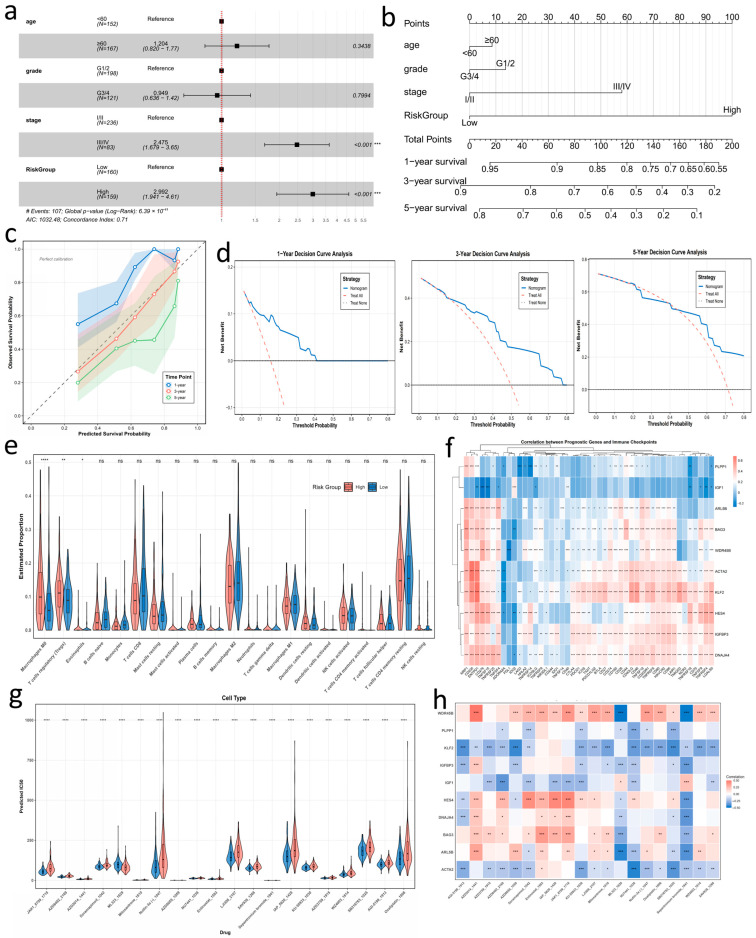
Multi-dimensional characterization of the *BAG3*-associated risk score. (**a**) Multivariable Cox regression showing hazard ratios and 95% confidence intervals for age, tumor grade, AJCC stage, and risk group. (**b**) Nomogram predicting 1-, 3-, and 5-year overall survival probabilities. (**c**) Calibration curves comparing predicted versus observed 1-, 3-, and 5-year overall survival probabilities. (**d**) Decision curve analysis comparing the nomogram with treat-all and treat-none strategies. (**e**) Violin plots of CIBERSORT-inferred immune cell fractions in high- and low-risk groups. (**f**) Heatmap of Spearman correlations between expression of the *BAG3*-associated (10 genes) and immune checkpoint molecules. (**g**) Violin plots of predicted drug sensitivity for the top 20 differential compounds between two risk groups. (**h**) Heatmap of Spearman correlations between expression of the BAG3-associated and drug sensitivity. * *p* < 0.05, ** *p* < 0.01, *** *p* < 0.001, **** *p* < 0.0001.

We therefore derived the final RS from the multivariable Cox model (see Methods for the formula). Patients were dichotomized at the cohort-specific median RS, and Kaplan–Meier analyses showed significant survival separation between the high- and low-RS groups in both TCGA-LIHC and ICGC-LIRI-JP (Figure 5f). In multivariable Cox models adjusted for age, tumor grade, and American Joint Committee on Cancer (AJCC) stage, RS remained independently associated with overall survival, with a hazard ratio (HR) of 2.992 for the high-RS group relative to the low-RS group (95% CI, 1.941–4.613; *p* = 6.88 × 10^−7^; Figure 6a). A nomogram integrating age, grade, AJCC stage, and dichotomized RS predicted 1-, 3-, and 5-year overall survival, with RS contributing the widest point range (Figure 6b). Calibration curves showed good agreement between predicted and observed survival, particularly at 3 years (Figure 6c), and decision curve analysis indicated net clinical benefit across a broad range of threshold probabilities (Figure 6d). Subgroup analyses stratified by age, grade, and AJCC stage further supported the robustness of the RS-based risk stratification (Supplementary Figure S8g,h).

After establishing the prognostic robustness of the RS, we further assessed the cellular context of the final signature genes in the discovery scRNA-seq dataset. At the broad cell-type level, most of these genes showed relatively high scaled expression in BAG3^+^ TAMs, although a few genes were also prominent in endothelial cells or fibroblasts. Within the myeloid compartment, the signature genes were largely enriched in BAG3^+^ TAMs compared with other myeloid subsets (Supplementary Figure S9a,b). Together, these patterns indicate that the final signature has a BAG3^+^ TAM-enriched but non-exclusive cellular context.

We further examined whether the *BAG3*-associated signal was tumor-enriched or also present in adjacent liver using 50 paired TCGA-LIHC tumor and adjacent non-tumor bulk RNA-seq samples. Tumor tissues showed higher *BAG3* expression and BAG3^+^ TAM marker-program scores than paired adjacent non-tumor liver, whereas the coefficient-weighted 10-gene RS formula score did not differ significantly between the two tissue types (Supplementary Figure S9c). In an etiology-adjusted sensitivity analysis using available TCGA-LIHC liver disease risk-factor annotations, the final Cox-based RS remained significantly associated with overall survival after additional adjustment for etiology/risk-factor category, with an HR of 2.929 for the high-RS group relative to the low-RS group (95% CI, 1.878–4.570; *p* = 2.17 × 10^−6^; Supplementary Table S6).

### 3.9. Immune and Therapeutic Associations of the BAG3-Associated Risk Score

Using CIBERSORT [34], we deconvoluted bulk RNA-seq data from the TCGA-LIHC cohort to estimate immune-cell fractions and compared them between high- and low-RS groups (Figure 6e). High-RS tumors showed higher inferred fractions of M0-like macrophages and regulatory T cells (Tregs) (M0: *p* < 0.0001; Tregs: *p* < 0.01), whereas the M1 polarization index [M1/(M1 + M2)] did not differ significantly between high- and low-RS groups (*p* = 0.88; Supplementary Figure S10a). Consistently, risk-associated genes (*BAG3*, *HES4*, *ARL5B*, *WDR45B*, and *IGFBP3*) were positively correlated with myeloid or regulatory populations, whereas protective genes (*KLF2*, *IGF1*, *PLPP1*, and *ACTA2*) showed the opposite trend and were positively associated with naïve B cells and memory CD4^+^ T cells (Supplementary Figure S10b). Together, these findings link higher RS to a myeloid- and regulatory-enriched immune context rather than to a classical M2-skewed macrophage shift.

We next correlated the 10 signature genes with 47 immune checkpoint molecules and ssGSEA-derived pathway activity scores for Hallmark and Reactome gene sets (Figure 6f). Risk-associated genes showed broad positive correlations with inhibitory and exhaustion-related checkpoints, including CD276/B7-H3, ENTPD1/CD39, TIGIT, and PD-1, whereas several protective genes showed weaker or opposite associations. High-RS tumors were enriched for proliferative programs, including G2M checkpoint, E2F targets, and MYC/MTORC1 signaling, whereas low-RS tumors were enriched for metabolic and homeostatic pathways linked to preserved hepatic function, including bile acid metabolism, xenobiotic metabolism, complement/coagulation pathways, and the KRAS signaling down signature (Supplementary Figure S10c,d).

To explore potential drug-response associations, we applied oncoPredict [36] to estimate the half-maximal inhibitory concentration (IC50) of 198 anticancer compounds in TCGA-LIHC tumors using Genomics of Drug Sensitivity in Cancer 2 [74] training data (Figure 6g,h; Supplementary Figure S10e). Low-RS tumors showed lower predicted IC50 values for several agents, including PAK inhibitors, paclitaxel, and DNA damage response inhibitors, whereas high-RS tumors exhibited higher predicted IC50 values for MDM2 and IAP inhibitors as well as selected JAK/PI3K/MTOR pathway inhibitors [75,76,77]. Gene–drug correlation analyses showed the same directionality, with *BAG3* and *WDR45B* correlating with higher predicted IC50 values, whereas *KLF2*, *ACTA2*, *PLPP1*, and *IGF1* were associated with lower predicted IC50 values for growth factor- and angiogenesis-related inhibitors.

## 4. Discussion

By integrating multiple datasets, we constructed a comprehensive single-cell and spatial atlas of HCC that identified major hepatic lineages and, through re-clustering of myeloid cells, identified five functionally distinct TAM subsets involved in immune modulation and matrix remodeling within the TME. Consistent with prior studies, four subsets broadly corresponded to established TAM phenotypes: MARCO^+^ TAMs showed homeostatic Kupffer-like features [10,45]; MT^+^ RTM− TAMs were enriched for lipid handling and xenobiotic detoxification [41,42,52]; MMP9^+^ TAMs displayed inflammatory and matrix-remodeling features [7,43]; and UBE2C^+^ TAMs represented a proliferative state [10,39]. In contrast, BAG3^+^ TAMs remain less well characterized in HCC [10], despite being noted in a pan-cancer myeloid cell atlas [39] and in colorectal cancer [46] based on heat shock chaperone expression. Functional enrichment analysis suggested that BAG3^+^ TAMs in HCC exhibit a *BAG3*-centered stress-remodeling program, characterized by enhanced chaperone-mediated proteostasis together with coordinated inflammatory and ECM/adhesion signatures, potentially contributing to an immunosuppressive microenvironment.

Pseudotime analysis traced TAMs to a VCAN^+^ monocyte origin that passed through a transitional state before diverging into two branches, with MARCO^+^ and BAG3^+^ TAMs enriched toward the terminal ends of their respective lineages. Because this analysis was based on cross-sectional scRNA-seq data and MMP9^+^ TAMs were underrepresented in the HBV-derived subset, the inferred trajectory should be interpreted as a representative transcriptional continuum rather than a complete lineage map of all TAM states in HCC. An exploratory non-HBV sensitivity analysis including MMP9^+^ TAMs showed that these cells did not form a clearly separated terminal branch, although interpretation remains limited by etiologic heterogeneity and sparse BAG3^+^ TAM representation. TAM states also varied across disease etiologies and tissue sites, with implications for therapeutic targeting. MARCO^+^ TAMs were mainly localized to adjacent non-tumor liver and were underrepresented in metastatic sites [52,64], suggesting that MARCO-directed interventions may preferentially act outside tumor sites and incompletely engage tumor-infiltrating TAMs [11]. In contrast, BAG3^+^ TAMs were enriched in tumor samples and were particularly prominent in HBV-related cases. These patterns suggest that tumor-enriched programs such as the BAG3^+^ TAM state may be particularly relevant to the immunosuppressive tumor niche, especially in HBV-associated HCC.

At the intercellular communication level, BAG3^+^ TAMs occupied a highly connected position in the inferred crosstalk network among parenchymal, stromal, and immune compartments. These cells showed relatively high inferred connectivity, particularly along the myeloid–hepatocyte axis, and integrated stress- and lipid-associated signals consistent with enhanced proteostasis and tissue remodeling. These differences related to hepatocyte state suggest that BAG3^+^ TAMs may integrate inflammatory inputs derived from malignant cells with stress or remodeling cues from the surrounding tumor bearing liver parenchyma, providing a plausible microenvironmental basis for their stress adaptive and immunoregulatory features. Among upstream stress-sensing cues converging on BAG3^+^ TAMs, the MIF–CD74 axis is particularly notable. Prior studies have implicated this pathway in immune regulation across multiple solid tumors [70,78,79] and in HCC progression through inflammatory macrophage populations in the liver [79], supporting its relevance in hepatic disease. In our analysis, receiver-side mapping suggested convergence on stress-responsive transcriptional circuits involving EGR1, NFKB1, AP-1, and FOXO3. This interpretation was further supported by SCENIC-inferred regulons, including ATF family members and MAZ, which connect stress-associated programs to angiogenic and immunomodulatory signaling. Exploratory spatial analyses provided contextual support for this framework, as BAG3^+^ TAM-enriched niches showed directionally concordant activity of these stress-responsive programs across tissue sections, supporting a spatially localized *BAG3*-centered remodeling niche rather than a diffuse global signal. Because the current ST data are spot-based rather than single-cell-resolved, they provide supportive spatial context but do not directly resolve specific sender–receiver pairs or proximal interaction patterns, or the exact cellular composition of *BAG3*-associated high-score spots. However, the spatial transcriptomics cohort was small and treatment-exposed, comprising four immune-enriched HCC sections obtained after neoadjuvant cabozantinib plus nivolumab, which may influence the observed macrophage states and inferred signaling patterns. Therefore, the observed *BAG3*-associated spatial patterns, inferred ligand–receptor interactions, and TF activity should be interpreted as supportive and hypothesis-generating rather than as definitive validation across HCC. These observations remain inferential and do not establish causal or directional signaling. CellChat and CellCall infer potential ligand–receptor and receptor–TF links from transcriptomic profiles, rather than directly demonstrating physical ligand–receptor engagement or functional signaling activity. Functional and spatial validation studies, such as macrophage-specific *BAG3* perturbation, macrophage–T cell co-culture assays, multiplex spatial profiling, and myeloid-targeted in vivo models, will be required to determine whether *BAG3*-associated stress programs contribute mechanistically to macrophage-mediated immune regulation in HCC. If validated, these pathways may provide a basis for future biomarker development and mechanistic studies.

To further evaluate the clinical associations of this program, we developed a *BAG3*-associated RS that showed stable prognostic discrimination. The reduced external performance of more flexible machine-learning models suggested possible cohort-specific fitting, whereas the Cox-based RS provided a more interpretable and stable formulation across the two bulk cohorts. The RS distinguished tumors with an immunosuppressive, hyperproliferative microenvironment enriched for M0-like macrophages, Tregs, and inhibitory checkpoints from tumors with a more metabolically balanced and T cell-competent niche. Because these immune-cell estimates were derived from bulk transcriptomic deconvolution, they should be interpreted as associations between the *BAG3*-associated risk score and immune contexture rather than evidence that BAG3^+^ TAMs directly induce T-cell suppression or macrophage polarization. The RS also aligned with predicted drug-response patterns, with low-RS tumors showing greater predicted sensitivity to cytoskeleton- and DNA repair-targeting agents and high-RS tumors showing reduced predicted sensitivity. Genes with positive RS coefficients were associated with stress adaptation, angiogenesis, ECM remodeling, and immune regulation, whereas genes with negative coefficients aligned with vascular quiescence, anti-inflammatory signaling, and matrix homeostasis. Because bulk tumor transcriptomes contain mixed cellular signals, the RS should not be interpreted as a direct measure of BAG3^+^ TAM abundance. Consistent with the scRNA-seq expression analysis, the final signature showed a BAG3^+^ TAM-enriched but non-exclusive cellular context, supporting its interpretation as a *BAG3*-associated program-level score. By capturing key transcriptional and microenvironmental features associated with the *BAG3*-associated TAM program, the RS remained independently associated with survival after adjustment for clinical covariates. Although BAG3^+^ TAMs showed preferential enrichment in primary tumors in the discovery dataset, the *BAG3*-associated program should not be interpreted as tumor-exclusive and may partly reflect background liver injury or inflammatory stress. Further validation in independent cohorts with matched tumor-adjacent bulk, single-cell or spatial profiles and detailed etiologic, metabolic, treatment, and histopathologic annotations will be required to more definitively separate tumor-associated and background-liver components.

## 5. Conclusions

In conclusion, our study delineates a BAG3^+^ TAM-associated program within the heterogeneous TAM landscape of HCC, characterized by stress adaptation, proteostasis dysregulation, stromal remodeling, and immunoregulatory features. These features are reflected in a *BAG3*-associated risk score with prognostic relevance. Together, our findings provide an integrated characterization of this myeloid-associated program in HCC and support its further evaluation in biomarker and mechanistic studies.

## Data Availability

The datasets analyzed in this study are publicly available. scRNA-seq data are available in GEO (https://www.ncbi.nlm.nih.gov/geo/, accessed on 30 September 2024) with accession numbers GSE149614 and GSE245906, and spatial transcriptomics data are available in GEO with accession number GSE238264. Bulk RNA-seq and clinical data were obtained from TCGA-LIHC (https://portal.gdc.cancer.gov, accessed on 30 September 2024) and ICGC-LIRI-JP (https://dcc.icgc.org, accessed on 30 September 2024). Processed supporting results are provided in the article and its Supplementary Materials.

## Supplementary Materials

The following supporting information can be downloaded at: https://www.mdpi.com/article/10.3390/genes17050562/s1, Supplementary Note S1: Detailed scRNA-seq data processing and integration; Supplementary Note S2: inferCNV-based malignant-cell classification; Supplementary Note S3: Additional computational details for downstream TAMs analyses; Supplementary Note S4: Additional details for spatial and bulk transcriptomic analyses; Supplementary Note S5: Expanded BAG3^+^ TAM-centered intercellular communication and downstream transcriptional circuits; Supplementary Note S6: Expanded spatial analyses of BAG3^+^ TAM-associated transcription factor activity; Supplementary Figure S1: Global structure and preprocessing overview of the discovery scRNA-seq dataset; Supplementary Figure S2: Construction and overview of the integrated validation scRNA-seq dataset: QC, integration, clustering, and annotation; Supplementary Figure S3: Myeloid subclustering and marker validation in the discovery scRNA-seq dataset; Supplementary Figure S4: Myeloid re-clustering and functional profiling in the integrated validation scRNA-seq dataset, highlighting the *BAG3*-enriched C7 cluster; Supplementary Figure S5: Exploratory pseudotime analysis of non-HBV-derived TAM subsets; Supplementary Figure S6: Panoramic view of cell communication and the BAG3^+^ TAM-centered network; Supplementary Figure S7: Bubble plot of ligand–receptor interactions involving BAG3^+^ TAMs in the non-HBV condition; Supplementary Figure S8: Extended multi-model evaluation of the 10-gene prognostic signature in HCC; Supplementary Figure S9: Cell-type expression patterns of the final *BAG3*-associated prognostic signature genes; Supplementary Figure S10: Immune, pathway and drug response correlates of the *BAG3*-associated risk score; Supplementary Table S1: Overview of discovery and integrated validation HCC scRNA-seq datasets; Supplementary Table S2: Spatial transcriptomic QC metrics for four HCC samples (GSE238264); Supplementary Table S3: Differential transcription factor activity in high and low BAG3^+^ TAM groups across multiple quantile thresholds; Supplementary Table S4: Prognostic association of 13 BAG3^+^ TAM marker genes identified by univariable Cox analysis (TCGA-LIHC); Supplementary Table S5: Feature importance comparison across three survival prediction models; Supplementary Table S6: Sensitivity analysis of the association between the final Cox-based RS and overall survival after adjustment for available etiology/risk-factor category; Supplementary Data S1: Differential expression results for five TAM subsets; Supplementary Data S2: Functional enrichment results for TAM subset marker genes; Supplementary Data S3: SCENIC regulon activity results across TAM subsets; Supplementary Data S4: inferCNV-based CNV scores and malignant-cell classification results; Supplementary Data S5: CellChat ligand–receptor interaction results; Supplementary Data S6: CellCall ligand–receptor–transcription factor analysis results.
