# Supplementary material for: Integrated Single-Cell and Spatial Transcriptomics Analyses Delineate a BAG3-Associated Macrophage Program with Microenvironmental and Prognostic Relevance in Hepatocellular Carcinoma
Source: Genes (Basel). 2026 May 11;17(5):562. doi: 10.3390/genes17050562 (PMC13206422; doi:10.3390/genes17050562)
Supplement: Supplementary file 1 [file genes-17-00562-s001.zip › genes-4280103-supplementary/genes-4280103-supplementary.zip!/genes-4280103-supplementary.pdf]

Supplementary Information for

Integrated Single-Cell and Spatial Transcriptomics Analyses Delineate a *BAG3*-Associated Macrophage Program with Microenvironmental and Prognostic Relevance in Hepatocellular Carcinoma

Ruixiang Zhang [1,†], Yifang Wei [1,†], Junda Yu [1], Yuansheng Li [1], Zuming You [1], Chenxi Xie [1], Siqi Xu [2,*] and Jiyuan Zhou [1,*]

[1] Department of Biostatistics, School of Public Health (State Key Laboratory of Multi-organ Injury Prevention and Treatment, and Guangdong Provincial Key Laboratory of Tropical Disease Research), Southern Medical University, Guangzhou 510515, China; zrx15751771690@i.smu.edu.cn (R.Z.); w12210152@i.smu.edu.cn (Y.W.); 3217042022@i.smu.edu.cn (J.Y.); li128795@i.smu.edu.cn (Y.L.); yzm886216@i.smu.edu.cn (Z.Y.); a22320064@i.smu.edu.cn (C.X.)

[2] Clinical Research Centre, School of Medicine, the Second Affiliated Hospital of South China University of Technology (Guangzhou First People's Hospital), Guangzhou 510180, China

[*] Correspondence: eyxsq@scut.edu.cn (S.X.); zhoujy@smu.edu.cn (J.Z.)

[†] These authors contributed equally to this work.

This file includes:

Supplementary Notes S1–S6;

Supplementary Figures S1–S10;

Supplementary Tables S1–S6.

Supplementary Note S1. Detailed scRNA-seq data processing and integration.

The discovery scRNA-seq dataset was obtained from a published study and included 21 samples from 10 patients with HCC across multiple tissue sites, including primary tumor, adjacent non-tumor liver, lymph-node metastasis, and portal vein tumor thrombus (PVTT). For validation, a paired tumor-adjacent scRNA-seq dataset from another published study was used, from which 10 paired tumor-adjacent cases (20 samples) were retained for analysis. These external paired samples were further combined with the paired samples from the discovery dataset to generate an integrated validation dataset comprising 18 paired tumor-adjacent cases (36 samples) from 18 patients. Clinicopathological characteristics of the discovery dataset and the integrated validation dataset are summarized in Supplementary Table S1.

For the discovery dataset, raw FASTQ files were aligned and quantified using Cell Ranger (v6.0.2; 10x Genomics, Pleasanton, CA, USA) with the GRCh38 reference genome under default parameters. The resulting gene expression matrices were analyzed in R (v4.4.0) using Seurat (v5.1.0). During quality control, cells with fewer than 500 or more than 8,000 detected genes, more than 50,000 unique molecular identifiers (UMIs), or over 15% mitochondrial gene content were excluded. After filtering, 70,749 high-quality cells were retained for downstream analyses. The filtered dataset showed a median of 4,491 UMIs and 1,470 detected genes per cell. For dimensionality reduction and clustering, 2,000 highly variable genes were identified using Seurat's FindVariableFeatures with the vst method, followed by principal component analysis (PCA) (Supplementary Figure. S1a, b). Batch effects across samples were corrected using Harmony (v1.2.1) through RunHarmony. A shared nearest-neighbor graph was constructed based on the top 30 principal components using FindNeighbors, and clusters were identified with FindClusters at a resolution of 1.0. t-distributed stochastic neighbor embedding (t-SNE) visualization was generated from the Harmony-corrected embeddings using RunTSNE. Cell-type annotation was performed using canonical markers; for refined lymphocyte annotations, $CD8^+$ T cells demonstrated cytotoxic potential (*CD8A/B* and *GZMK*), $CD4^+$ T cells exhibited naïve/memory signatures (*IL7R* and *CCR7*), Tregs expressed lineage-defining

transcription factors *FOXP3* together with inhibitory receptors *CTLA4*, and NK cells displayed characteristic activation markers (*KLRD1*, *NCR1*, and *FCGR3A*).

To further characterize myeloid heterogeneity, myeloid-lineage cells were subsetted according to the major-lineage annotation and re-clustered using the same analytical framework. The clustering resolution was set to 0.3 based on clustree-assessed stability and interpretability (Supplementary Figure. S3a). Marker genes for each myeloid subcluster were identified using Seurat FindAllMarkers under the predefined differential-expression thresholds. Subset annotation was performed based on canonical myeloid markers, cluster-level marker enrichment, and functional profiles, rather than by applying single-gene expression thresholds (Supplementary Figure. S3b, c). Accordingly, the label "BAG3$^+$ TAMs" denotes a *BAG3*-enriched TAM cluster characterized by a broader stress-chaperone and matrix-remodeling transcriptional program, rather than a binary *BAG3*-positive cell population. In addition to the five TAM subsets, the myeloid compartment also included three dendritic cell (DC) subsets, VCAN$^+$ monocytes, and sparse mast cells. Specifically, CLEC9A$^+$ cDC1 were supported by expression of *CLEC9A* and *XCR1*, CD1E$^+$ cDC2 by *CD1E*, *CD1C*, and *CLEC10A*, and LILRA4$^+$ pDC by *LILRA4* and *JCHAIN*. VCAN$^+$ monocytes were characterized by *VCAN* and *S100A8/A9*, together with chemokine-associated genes including *CCL3/4* and *CXCL2/8*. The sparse mast cell population was identified by expression of *TPSAB*, *CPA3*, and *HDC*. These marker genes were used as supportive evidence for lineage annotation of the non-TAM myeloid populations.

For paired tumor-adjacent validation analyses, the integrated validation dataset was normalized and analyzed using the same general framework, with cross-dataset integration performed using Seurat's IntegrateLayers() and additional batch correction implemented with Harmony. Clustering of the integrated validation dataset was performed at a resolution of 1.2 to obtain finer cell-state granularity. In the integrated validation dataset, myeloid-specific re-clustering identified a distinct *BAG3*-enriched cluster (C7) characterized by stress-chaperone transcripts, including *BAG3*, *HSPA6*, *HSPH1*, *HSPD1*, *HSPE1*, and *DNAJB4*, together with monocyte/macrophage-associated markers such as *CD68*, *VCAN*, and *MRC1*.

Supplementary Note S2. inferCNV-based malignant-cell classification.

Large-scale CNVs were inferred using inferCNV (v1.22.0). T/NK cells, myeloid cells, endothelial cells, fibroblasts, and B cells were used as reference populations, and hepatocytes were treated as the observed population. Genes were ordered by genomic coordinates and analyzed with cutoff = 0.1, cluster_by_groups = TRUE, and HMM = TRUE. This yielded a continuous CNV matrix $\boldsymbol{X}$ (genes × cells). For each cell c, a CNV amplitude score was defined as:

$$S1_c = \frac{1}{G}\sum_{g=1}^{G}\left|X_{gc} - center_g\right|$$

where G is the number of genes and $center_g$ denotes the median CNV value of gene g across reference cells (i.e., the neutral baseline under the inferCNV output scale). Malignant cells were classified using a reference-anchored threshold:

$$t_{S1} = \text{median}(S1_{\text{ref}}) + 3 \times \text{MAD}(S1_{\text{ref}})$$

where $S1_{\text{ref}}$ denotes the collection of $S1$ score computed across all reference cells, and MAD denotes the median absolute deviation. Cells exhibiting $S1_c > t_{S1}$ were classified as malignant, and the remaining cells were designated non-malignant. This reference-anchored strategy limits false positives among non-tumor lineages while retaining sensitivity to chromosome-arm-scale alterations detected under the HMM-enabled inferCNV framework. The subsequent differential expression analysis between inferCNV-defined malignant and non-malignant hepatocytes was treated as a descriptive comparison and did not explicitly adjust for etiologic covariates.

Supplementary Note S3. Additional computational details for downstream TAMs analyses.

TAM context preference analysis

The preference of TAM subsets for HCC etiologies and tissue regions was quantified using the STARTRAC-dist index, as described by Zhang et al. For each TAM subset and context, we constructed a contingency table of observed cell counts and applied a chi-squared test to assess the deviation from random expectation. The STARTRAC-dist index was computed by first defining the observed count $O_{s,c}$ and the expected count $E_{s,c}$ under the independence assumption, and then calculating $R_{o/e} = O_{s,c}/E_{s,c}$, where expected counts were derived under the independence assumption. Values of $R_{o/e} > 1$ indicate enrichment, whereas those of $R_{o/e} < 1$ means depletion.

Pseudotime trajectory inference

Pseudotime analysis of HBV-derived myeloid cells, in which BAG3^{+} TAMs were sufficiently represented to allow robust trajectory inference, was performed using Monocle2 (v2.32.0). VCAN^{+} monocytes and TAMs were subset to construct the trajectory after filtering lowly expressed genes. The ordering gene set combined highly variable genes and those differentially expressed across TAM subsets. Dimensionality reduction used DDRTree (max_components = 2). In Monocle2, pseudotime direction is defined relative to a designated root state rather than being assigned automatically as a biologically oriented lineage. Here, VCAN^{+} monocytes were specified as the root state because they showed monocyte-like and relatively less differentiated features, which is biologically consistent with the commonly proposed monocyte-to-macrophage progression framework in TAM development. Genes significantly associated with pseudotime were clustered into four temporal modules using ClusterGVis (v0.1.4), followed by GO-BP enrichment analysis (FDR < 0.05). MMP9^{+} TAMs were excluded due to underrepresentation in HBV samples. Accordingly, the arrows shown in Figure. 3e–f indicate inferred progression from this designated root state and do not represent direct lineage tracing.

SCENIC, CellChat, and cellcall analyses

Gene regulatory activity in TAMs was quantified using SCENIC (v1.3.1). TF-target gene co-expression modules were inferred using the GENIE3 algorithm, followed by motif enrichment analysis with RcisTarget to define regulons. AUCell was then used to calculate regulon activity scores (RAS) at the single-cell level. To evaluate the cell-type specificity of each regulon, we calculated the regulon specificity score (RSS) following the method described by Suo et al. Briefly, probability distributions of RAS and cell identity were compared using Jensen–Shannon divergence (JSD), and RSS was computed as: $RSS(R, C) = 1 - \sqrt{JSD(P^R, P^C)}$, P^R and P^C represent the normalized distributions of RAS and cell-type labels, respectively. A higher RSS indicates stronger specificity of the regulon to a particular cell-type.

CellChat (v2.2.0) was used to infer intercellular communication based on Seurat-normalized expression values, curated cell-type annotations, and the built-in human ligand–receptor database CellChatDB.human. Overexpressed ligands, receptors, and ligand–receptor pairs were identified using the standard CellChat workflow. Communication probabilities were estimated using computeCommunProb(type = "triMean", raw.use = TRUE, population.size = FALSE), followed by filterCommunication(min.cells = 5). The subsetData quantile parameter was set to 0.90. Interactions with permutation-based $P<0.05$ were retained for visualization and summary. Network-level communication patterns were visualized using bubble plots and circle plots. For BAG3$^+$ TAMs, bidirectional bubble plots were used to display the top inferred interactions for each sender–receiver group.

Because BAG3$^+$ TAMs were sparsely represented in non-HBV tumors, detailed BAG3$^+$ TAM-centered signaling analyses were performed primarily in HBV-derived samples. The non-HBV results are presented as reference analyses in Supplementary Figure. S6e and Supplementary Figure. S7, with corresponding ligand–receptor interaction lists for both HBV and non-HBV conditions provided in Supplementary Data S5.

Downstream transcriptional coupling was assessed using CellCall (v1.0.7), which links ligand–receptor interactions to receptor-side transcription factor activity using a

curated receptor–TF prior. Ligand–receptor and receptor–TF links were retained using the minimum-expression criterion implemented in the CellCall workflow, with min.expression = 0.9. Significant interactions were retained at FDR<0.05. LR scores were calculated to quantify inferred ligand–receptor communication strength, and downstream TF activity was mapped to identify potential transcriptional programs associated with the inferred signaling axes. Sankey plots and ridge plots were used to visualize representative ligand–receptor–TF circuits, and TF-level GSEA was used as a complementary analysis to assess transcription factor enrichment.

Supplementary Note S4. Additional details for spatial and bulk transcriptomic analyses.

Spatial transcriptomics processing and multi-algorithm spatial scoring

We analyzed four immune-enriched HCC samples (ST01-R, ST02-R, ST03-R, and ST04-NR) from the spatial transcriptomics dataset GSE238264 obtained from the NCBI Gene Expression Omnibus database (https://www.ncbi.nlm.nih.gov/geo/), which was generated using the Illumina NovaSeq 6000 platform. After QC and normalization with SCTransform, PCA (40 PCs) and clustering were performed for each section independently. Clustering resolutions were set to 0.3 (ST01-R), 0.5 (ST02-R), 0.9 (ST03-R), and 0.5 (ST04-NR). Detailed spot-level QC metrics, including median detected genes and UMI counts per section, are summarized in Supplementary Table S2. Cell types were annotated based on canonical marker expression and spatial distribution patterns, thereby identifying immune cells (*CD3D*, *CD79A*, *CD68*, and *NCAM1*), stromal components (*VWF*, *ACTA2*, *COL1A1*, and *DCN*), multiple hepatocyte populations (*ALB*, *PDIA3*, *CYP1A2*, *CP*, *PRSS1*, etc.), and a cholangiocyte-like compartment marked by KRT7 and DKK1. Necrotic regions and cell types not relevant to downstream analyses were excluded. Gene-set activity was evaluated using AUCell (v1.26.0), UCell (v2.9.0), and Seurat::AddModuleScore. To improve robustness, the three scores were integrated using robust z-score normalization based on the median and median absolute deviation, followed by equal-weight averaging to generate a composite metaZ score. Cells or spatial spots were stratified into high- and low-score groups using the 75th, 80th, and 85th percentiles. Between-group comparisons were performed using two-sided Wilcoxon tests with FDR correction, and effect sizes were quantified using Cliff's δ.

Bulk RNA-seq data processing, prognostic modeling, and leakage-control procedures

Bulk RNA-seq expression profiles and clinical annotations for the TCGA-LIHC cohort were downloaded via the GDC API. The TCGA-LIHC dataset contains primary hepatocellular carcinoma samples as well as a limited number of adjacent non-tumor liver tissues. In this study, analyses were restricted to primary tumor samples with

complete overall survival and covariate information. Genes detected in fewer than 50% of tumor samples were removed. Gene expression values, quantified as transcripts per million (TPM), were transformed using $\log_2(\mathrm{TPM}+1)$. Survival data were quality-controlled by excluding cases with overall survival < 30 days, removing samples with missing survival time or status, and converting survival time from days to months. For clinical-adjusted analyses, samples with missing age, tumor grade, or AJCC stage were further excluded so that feature screening, model fitting, and clinical association analyses used a consistent complete-case TCGA-LIHC training set.

The external cohort ICGC-LIRI-JP was obtained from the ICGC data portal. This cohort comprises hepatocellular carcinoma patients with RNA-seq and overall survival data, and primary tumor samples with complete survival information were processed using the same gene-filtering criteria, normalization strategy and survival quality-control steps as for TCGA-LIHC to ensure comparability between cohorts. Gene symbols were harmonized where necessary, and expression values were transformed to the $\log_2(\mathrm{TPM}+1)$ scale when required. ICGC-LIRI-JP was used only for external evaluation after the final genes and models had been defined.

Candidate genes were defined as the top 50 upregulated marker genes of BAG3+ TAMs ranked by average log2 fold change in the single-cell analysis. These genes were intersected with the TCGA-LIHC bulk expression matrix. Univariate Cox proportional hazards models were fitted in TCGA-LIHC, and genes with nominal $p < 0.05$ (no multiple-testing correction) were retained. This univariable Cox filtering step was used only as an initial dimension-reduction step within the predefined BAG3+ TAM marker-gene set and was not interpreted as the final feature-selection procedure. Genes passing this initial screen were then entered into an elastic-net Cox model using the glmnet package (v4.1-8), with family = "cox", alpha = 0.5, 10-fold cross-validation, and a fixed random seed of 10,210. The lambda value that minimized cross-validated partial-likelihood deviance, lambda.min, was selected, and genes with non-zero coefficients at lambda.min defined the final 10-gene *BAG3*-associated prognostic signature.

Using these 10 genes as predictors, four survival models were fitted in TCGA-LIHC: multivariable Cox regression, random survival forest (RSF), XGBoost Cox

survival model, and survival support vector machine (SVM). The multivariable Cox proportional hazards model was implemented using the survival package (v3.5-8), with Efron's method for ties. The RSF model was constructed using randomForestSRC (v3.4.1), with ntree = 400, mtry = floor(sqrt(p)), nodesize = 30, nsplit = 10, and variable importance estimation enabled, where p denotes the number of signature genes. The XGBoost model was fitted using xgboost (v1.7.11.1) with objective = "survival:cox" and eval_metric = "cox-nloglik". The hyperparameters were eta = 0.05, max_depth = 2, subsample = 0.7, colsample_bytree = 0.7, min_child_weight = 5, lambda = 2, and alpha = 0.5. The number of boosting iterations was selected by five-fold cross-validation with nrounds = 500 and early_stopping_rounds = 20. The survival SVM model was fitted using survivalsvm (v0.0.6) with standardized features, an RBF kernel, gamma.mu = 0.01, diff.meth = "makediff1", and type = "vanbelle2".

To avoid information leakage, all feature screening, elastic-net selection, model fitting, hyperparameter tuning, and training-set scaling were performed within TCGA-LIHC. ICGC-LIRI-JP was not used for feature selection, elastic-net tuning, model training, coefficient estimation, hyperparameter optimization, or marker-gene selection. For XGBoost and SVM, feature standardization parameters were estimated in TCGA-LIHC and then applied unchanged to ICGC-LIRI-JP.

Each model produced a patient-specific risk score (linear predictor or predicted log-risk). For the Cox model, the RS was computed as: RS=$\sum_{i=1}^{n} \beta_i \times log_2(\mathrm{TPM}_i + 1)$, where $n = 10$ denotes the number of signature genes, β_i represents the regression coefficient, and $log_2(\mathrm{TPM}_i + 1)$ denotes the normalized expression value of gene i. Patients were stratified by the cohort-specific median RS for Kaplan-Meier visualization. C-index and time-dependent ROC analyses were performed using continuous model scores and did not depend on the dichotomized risk-group labels. Kaplan-Meier analyses were performed using the R package survival (v3.5-8) and survminer (v0.5.0), with significance assessed by the log-rank test ($p < 0.05$). Model performance was evaluated by Harrell's C-index and time-dependent ROC curves at 1-, 3-, and 5-year with timeROC (v0.4). To assess gene contributions, we quantified feature importance in the multivariable Cox, RSF, and XGBoost models using their native

importance measures, including absolute coefficient magnitude for Cox, variable importance for RSF, and gain-based feature importance for XGBoost. The resulting gene-importance rankings were compared across models (Figure. 5e). Feature robustness was assessed using the 10-fold cross-validated elastic-net selection procedure, cross-model feature-importance comparisons, and external evaluation in ICGC-LIRI-JP. We did not interpret these analyses as formal stability selection, and further validation in independent cohorts will be required to assess feature-selection stability across datasets and platforms. A nomogram integrating age, tumor grade, AJCC stage, and RS was evaluated by bootstrap calibration (B = 300) and decision curve analysis.

Given its more stable cross-cohort C-index and coefficient-based interpretability, the multivariable Cox model was selected for final RS construction. All downstream clinical analyses, including multivariable Cox regression, nomogram construction, calibration, decision curve analysis, subgroup analysis, and etiology-adjusted sensitivity analysis, were performed using this final Cox-based RS.

For paired tumor-adjacent bulk analysis, available TCGA-LIHC patients with both primary tumor and adjacent non-tumor RNA-seq samples were identified using the first 12 characters of the TCGA barcode. Paired comparisons were performed for *BAG3* expression, the coefficient-weighted 10-gene RS formula score, and the $BAG3^{+}$ TAM marker-program score using paired Wilcoxon signed-rank tests. P values were adjusted using the Benjamini–Hochberg method across the three comparisons.

For etiology-adjusted sensitivity analysis, liver disease risk-factor annotations were derived from the TCGA-LIHC field "history_hepato_carcinoma_risk_factors" and grouped as HBV, HCV, and NBNC/Other. Samples containing hepatitis B were classified as HBV; samples containing hepatitis C but not hepatitis B were classified as HCV; all remaining samples were grouped as NBNC/Other, including alcohol-related, NAFLD-related, no documented primary risk factor, other, and unavailable/blank annotations. This etiology/risk-factor category was added to the multivariable Cox model together with age group, tumor grade group, AJCC stage group, and the final Cox-based RS. HRs for the RS variable were reported to evaluate whether the

prognostic association remained after etiologic adjustment, whereas category-specific etiology coefficients were included only for adjustment and were not interpreted mechanistically.

Immune, drug-response, and pathway analyses

Immune infiltration was estimated using CIBERSORT with the LM22 signature matrix, 1,000 permutations, and quantile normalization enabled, using $log_2(\mathrm{TPM}_i + 1)$ as input. Immune checkpoint activity was quantified by ssGSEA using a 47-gene checkpoint set. Drug sensitivity was predicted using oncoPredict with Genomics of Drug Sensitivity in Cancer version 2 training data, yielding sample-specific half-maximal inhibitory concentration estimates across 198 compounds. Pathway activity was quantified by ssGSEA using Reactome and Hallmark gene sets from the Molecular Signatures Database, and associations with RS were assessed by correlation analysis and comparisons between high- and low-risk groups. All statistical tests were two-sided, with Benjamini-Hochberg correction applied where appropriate.

Supplementary Note S5. Expanded BAG3$^+$ TAM-centered intercellular communication and downstream transcriptional circuits.

To further dissect the BAG3$^+$ TAM-centered interaction network summarized in the main text, we examined the detailed sender–receiver architecture and downstream transcriptional circuits at higher resolution. Based on the refined T/NK-cell annotation shown in Figure. 3j, k, CellChat analysis indicated that BAG3$^+$ TAMs occupied a highly connected position within the multicellular network, with broad interactions involving parenchymal, stromal, and immune compartments (Figure. 4c–e; Supplementary Data S5).

Incoming signals to BAG3$^+$ TAMs were inferred primarily from tumor, endothelial, and fibroblast populations. In addition to the TREM2–TYROBP complex highlighted in the main text, its predicted upstream ligands included APOE, APP, APOA1, and APOA2, supporting a microenvironmental context enriched for lipid-associated and immunomodulatory cues. BAG3$^+$ TAMs also received MIF–CD74-related inputs, with or without CXCR4/CD44, consistent with inflammatory survival signaling, NF-κB/MAPK activation, and cytokine responses. Another recurrent incoming route was PPIA–BSG, which has been associated with extracellular matrix reorganization and vascular permeability. Together, these results suggest that BAG3$^+$ TAMs preferentially sense a combination of lipid-associated, inflammatory, and stress-responsive signals in the local niche.

Outgoing signaling from BAG3$^+$ TAMs was directed mainly toward stromal and immune compartments. Toward endothelial cells and fibroblasts, the dominant routes included VEGFA–KDR/FLT1 together with integrin- and collagen-associated adhesion modules, including VTN–ITGAV/ITGB1 and COL4A1–ITGA1/ITGB1. Additional stromal-facing signals included PPIA–BSG and IGFBP3–TMEM2, further supporting roles in vascular remodeling and stromal activation. Toward tumor cells, BAG3$^+$ TAMs were predicted to communicate through PPIA–BSG, LGALS9–P4HB, and collagen-associated interactions involving SDC/CD44, consistent with matrix anchoring and survival-related signaling. Toward lymphocytes, the major BAG3$^+$ TAM-centered axes included CXCL12–CXCR4, MIF–CD74-related signaling involving CXCR4/CD44,

and HLA-E–CD94/NKG2A, supporting functions in lymphocyte recruitment, retention, and effector regulation. Beyond BAG3$^+$ TAM–lymphocyte communication, several signaling routes recurred across immune sender–receiver configurations. In particular, MIF–CD74 and HLA-II–CD4 signaling were repeatedly observed in configurations involving BAG3$^+$ TAMs and other immune populations, including CLEC9A$^+$ cDC1, CD1E$^+$ cDC2, and LILRA4$^+$ pDC subsets (Supplementary Figure. S6f). This recurrence suggests that these pathways are broadly used within the immune compartment and may contribute to coordinated antigen-related and immunoregulatory signaling within the HCC microenvironment.

To connect these ligand–receptor interactions with intracellular responses, we further examined CellCall results (Supplementary Data S6). BAG3$^+$ TAMs showed increased activity of target genes associated with EGR1, NFKB1, AP-1 components JUN/JUND and FOS, together with additional enrichment of FOXO3 and JUNB (Figure. 4f). Ridge-density distributions of these target-gene activity scores were shifted toward higher values relative to the global myeloid background, supporting selective activation of these transcriptional circuits in BAG3$^+$ TAMs. These TF-associated programs were consistent with immediate-early, inflammatory, and stress-balancing responses, in line with the stress-adaptive phenotype of this subset.

Gene Set Enrichment Analysis (GSEA) of these TF-associated target-gene programs further confirmed coordinated upregulation, with positive normalized enrichment scores at $FDR < 0.05$ (Figure. 4g). The enriched programs broadly mapped to functions related to antigen presentation, vascular remodeling, chemotaxis, extracellular matrix organization, and lysosomal processes, supporting the interpretation that BAG3$^+$ TAMs couple stress signaling to microenvironmental remodeling and immune regulation. Sankey diagrams provided an integrated view of BAG3$^+$ TAMs as both receivers and senders within this network (Figure. 4h–k). As receivers, BAG3$^+$ TAMs integrated chemotactic and survival cues through CCL3/4/5–CCR1 and CSF1–CSF1R, together with inflammatory and regulatory inputs through IFNG–IFNGR1/2 and TGFβ–TGFBR1/2. These incoming routes converged on transcriptional programs centered on NF-κB, AP-1 (JUN/FOS), and FOXO3, with

additional contributions from RB1. As senders, BAG3$^+$ TAMs transmitted proangiogenic, matrix-remodeling, and migratory outputs toward endothelial and fibroblast compartments through VEGFA–KDR/FLT1, AREG/EREG/HBEGF–EGFR, PDGFB/C–PDGFRA/B, and TGFβ–TGFBR1/2. In parallel, BAG3$^+$ TAMs were predicted to modulate T- and NK-cell recruitment and effector states through MIF–CD74-related signaling, CXCL12–CXCR4, and HLA-E–CD94/NKG2A. Taken together, these expanded analyses support an inferred model in which BAG3$^+$ TAMs occupy a highly connected position in a stress-adaptive and immunoregulatory communication network in HCC. Specifically, they are predicted to integrate lipid-associated and inflammatory survival cues from the surrounding microenvironment, activate transcriptional circuits centered on NF-κB, AP-1, and FOXO3, and show potential outputs associated with vascular remodeling, extracellular matrix reorganization, and regulation of lymphocyte function.

Supplementary Note S6. Expanded spatial analyses of BAG3$^+$ TAM-associated transcription factor activity.

To further document the section- and threshold-specific patterns underlying the spatial analyses, immune regions in each section were stratified according to the BAG3$^+$ TAM metaZ score, and inferred transcription factor (TF) activity was compared between BAG3$^+$ TAM-high and BAG3$^+$ TAM-low groups under multiple upper-quantile cutoffs. Complete statistics for all sections, cutoffs, and TFs are provided in Supplementary Table S3.

Across sections, AP-1 and EGR1 showed the most stable positive differences in BAG3$^+$ TAM-high regions. In ST01-R and ST03-R, both TFs remained significant across the 75th, 80th, and 85th percentile cutoffs. In ST02-R, EGR1 remained significant across cutoffs, whereas AP-1 reached significance at selected thresholds. In ST04-NR, both TFs again showed clear positive differences, and the corresponding spatial activity maps supported concordant enrichment in BAG3$^+$ TAM-high regions (Figure. 4l). Detailed statistics for the ST04-NR example shown in the main text are listed in Supplementary Table S3. Compared with AP-1 and EGR1, NFKB1 showed more variable but still broadly supportive spatial patterns. Significant increases were observed under multiple upper-quantile cutoffs in ST01-R, ST03-R, and ST04-NR, whereas the effects in ST02-R remained directionally positive but were not consistently significant after multiple-testing correction. FOXO3 showed moderately consistent positive differences, reaching significance under most upper-quantile cutoffs in ST01-R and ST04-NR, but showing weaker or less stable significance in ST02-R and ST03-R. These patterns indicate that NFKB1 and FOXO3 contribute supportive, but less uniformly stable, spatial signals relative to AP-1 and EGR1. By contrast, RB1 showed no consistent spatial association with BAG3$^+$ TAM enrichment. Most comparisons across sections and cutoffs did not reach significance, indicating that RB1 is unlikely to represent a core spatial correlate of BAG3$^+$ TAM-high regions. This pattern suggests that, although RB1 appeared in the broader receiver-side transcriptional circuitry inferred by CellCall, it was not robustly supported at the spatial level. Effect sizes, measured by Cliff's δ, generally ranged from 0.14 to 0.33, corresponding to small-to-

moderate effects. Although the magnitude of these effects was modest, their direction was largely concordant across sections and cutoffs, supporting the robustness of the overall spatial trend. In the violin plots and bar plot shown for ST04-NR (Figure. 5a, b), BAG3^{+} TAM-high regions were defined as the top 15% spatial spots ranked by the BAG3^{+} TAM metaZ score. Taken together, these section- and cutoff-specific analyses support the robustness of the spatial TF patterns summarized in the main text and provide detailed statistical support for the BAG3^{+} TAM-associated stress and inflammatory remodeling program.

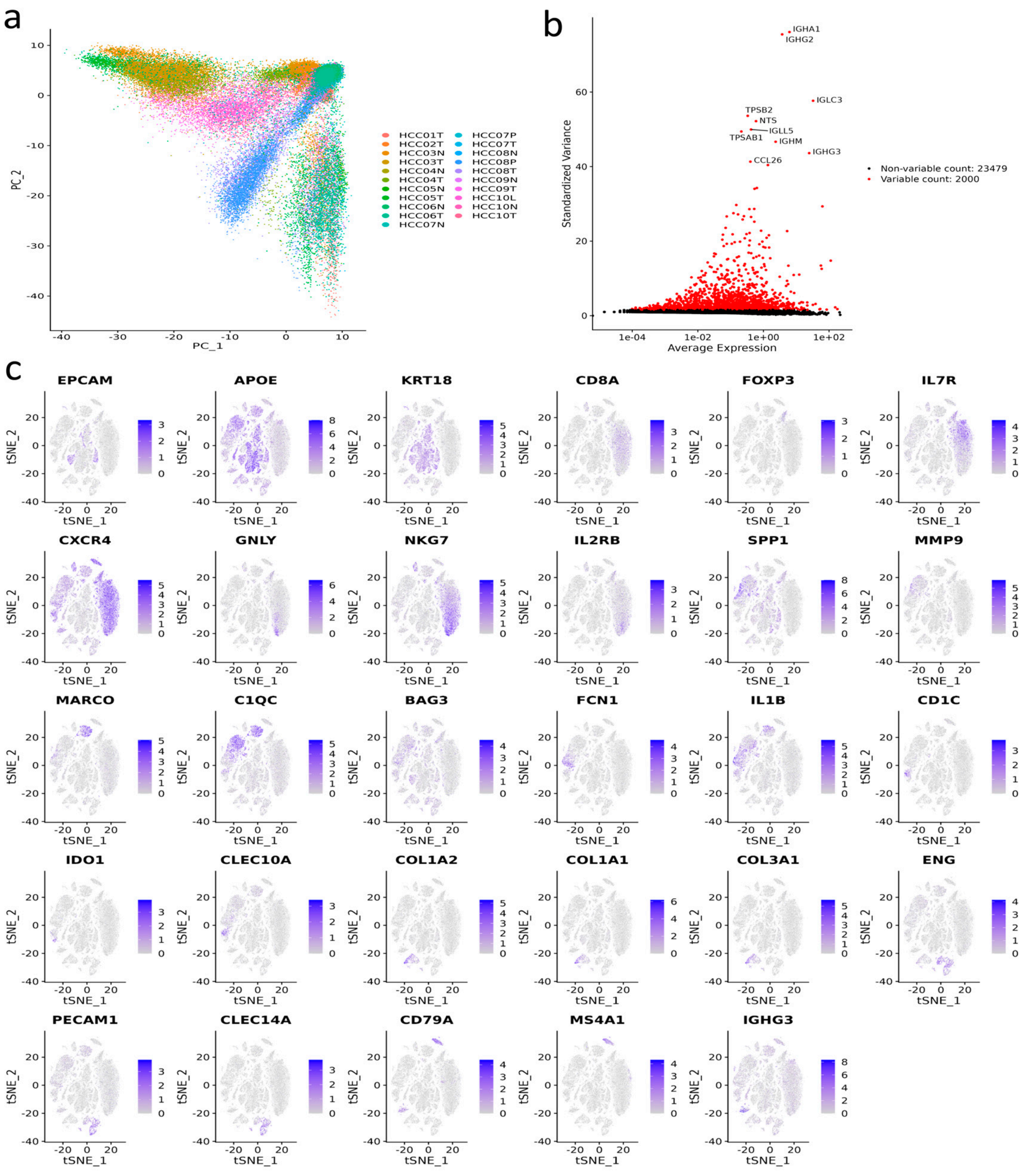

Supplementary Figure S1. Global structure and preprocessing overview of the discovery scRNA-seq dataset. (a) Principal component analysis of all QC-passed cells (PC1 and PC2), colored by sample; each point represents one cell, illustrating inter-sample heterogeneity and partial overlap. **(b)** Mean–variance relationship used to select highly variable genes (mean expression and standardized variance); red points indicate the 2,000 highly variable genes retained for downstream PCA, and the top 10 genes are labeled. **(c)** Extended feature plots of additional canonical markers used for cell-type annotation on the t-SNE; color indicates log-normalized expression.

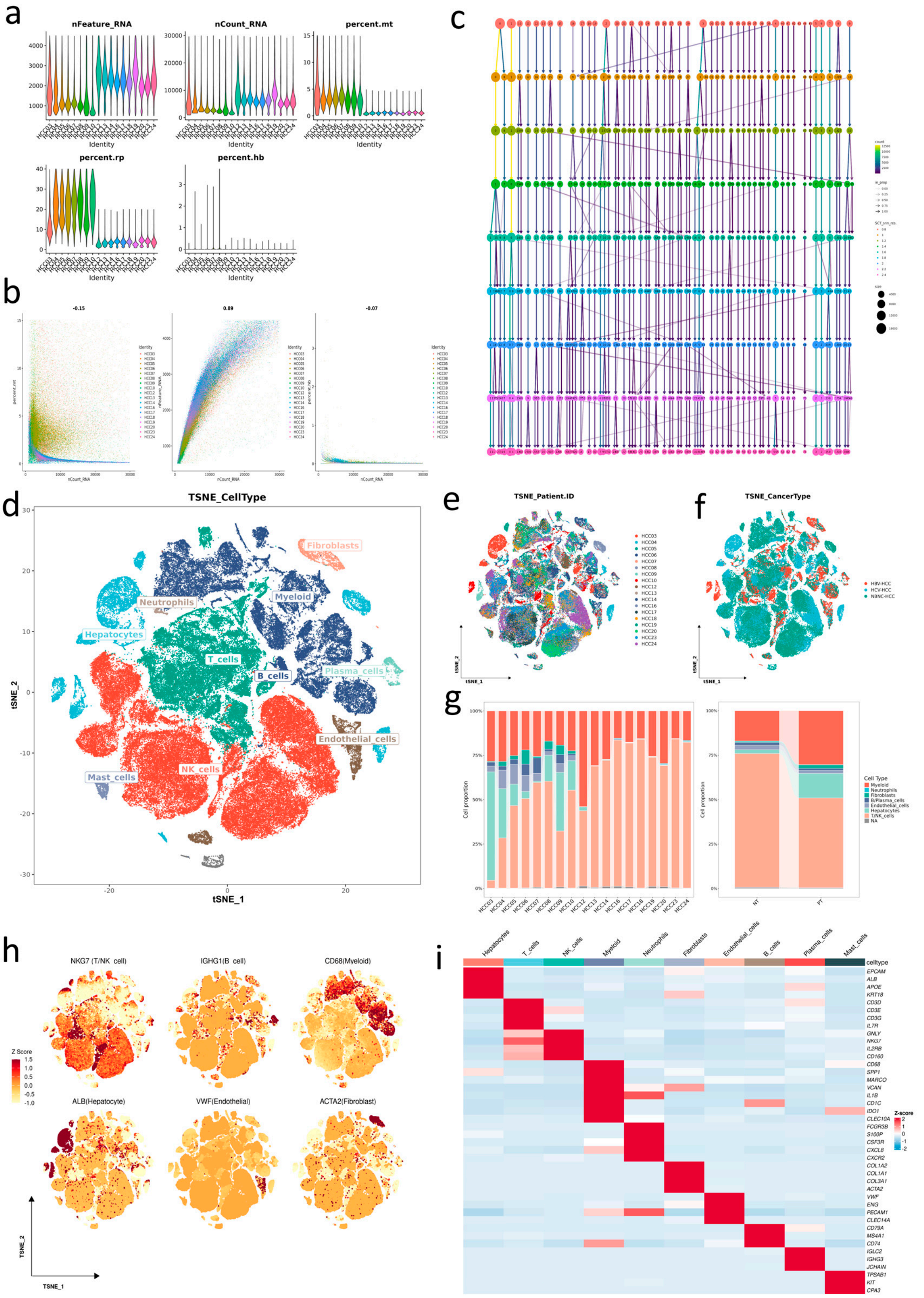

Supplementary Figure S2. Construction and overview of the integrated validation scRNA-seq dataset: QC, integration, clustering, and annotation. (a) Violin plots showing the distributions of standard per-cell QC metrics across patients, including the number of detected genes (nFeature_RNA), total UMI counts (nCount_RNA), and the

fractions of mitochondrial (percent.mt), ribosomal (percent.rp), and hemoglobin (percent.hb) transcripts. (b) Pairwise scatter plots of key QC metrics after filtering, showing relationships between nCount_RNA and percent.mt (left), nCount_RNA and nFeature_RNA (middle), and nCount_RNA and percent.hb (right). Each point represents one cell and is colored by patient; numbers denote the Pearson correlation coefficient, illustrating typical depth-associated (nCount_RNA and nFeature_RNA) and depth-independent patterns. (c) Clustree plot summarizing clustering structure across resolutions to determine the optimal resolution (1.2) after dataset integration and batch correction (see the Methods section). (d) t-SNE of the integrated dataset colored by annotated cell types. (e) t-SNE colored by patient, showing mixing across individuals. (f) t-SNE colored by etiology (HBV, HCV, and NBNC-HCC). (g) Stacked bar plot summarizing cell-type composition by patient and comparing compositions between PT (primary tumor) and NT (adjacent non-tumor liver) samples. (h) Feature plots of canonical markers (NKG7, IGHG1, CD68, ALB, VWF, and ACTA2) supporting cell-type annotation. (i) Heatmap of marker genes across annotated cell types (row Z-score of mean expression).

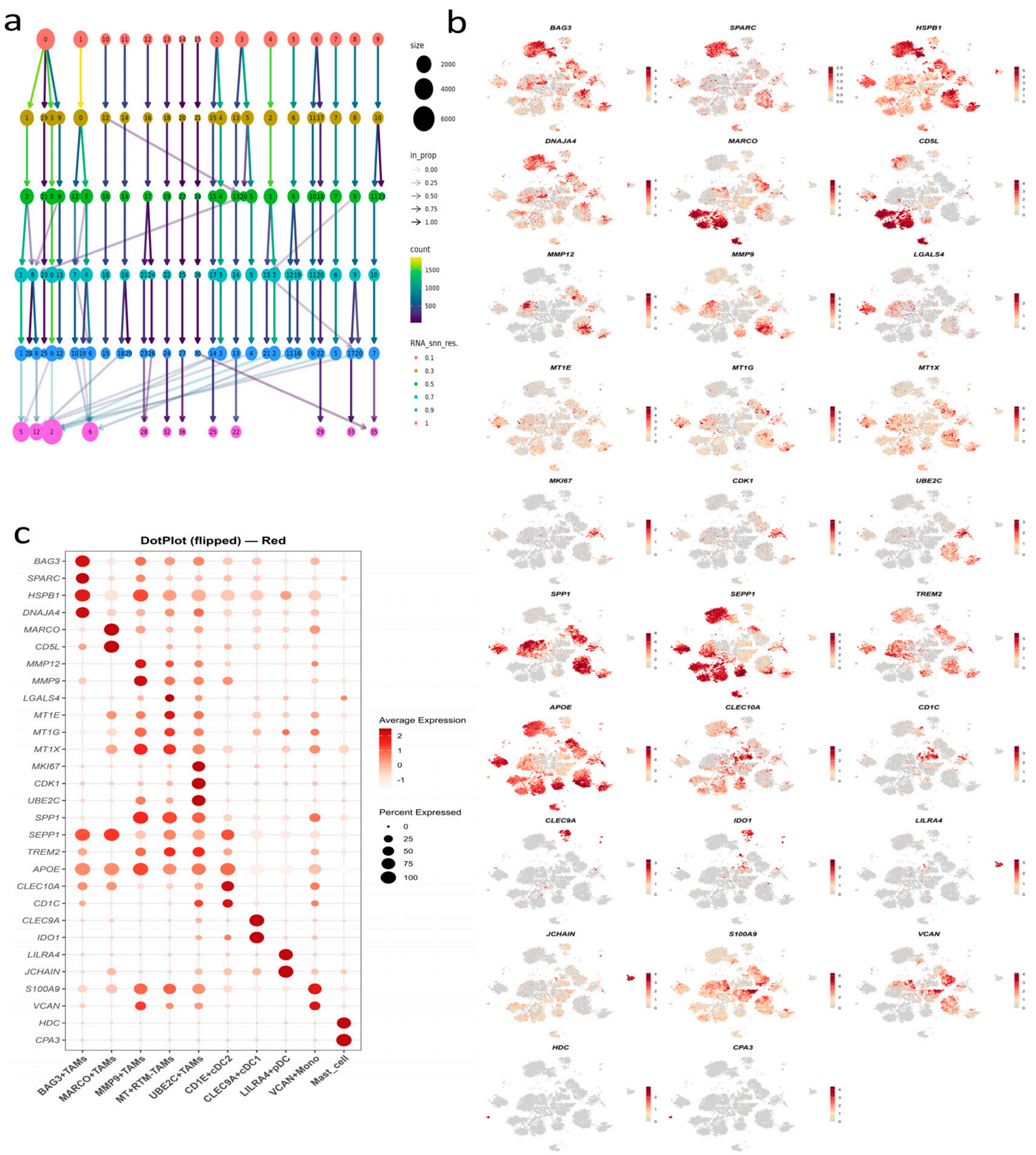

Supplementary Figure S3. Myeloid subclustering and marker validation in the discovery scRNA-seq dataset. (a) Clustree plot for myeloid-lineage cells showing cluster stability and inheritance across resolutions; node size indicates cell number and edges indicate cluster correspondence. Resolution 0.3 was selected for downstream myeloid subclustering based on stability and interpretability. **(b)** Feature plots of representative myeloid markers and previously reported TAM-state markers, including *BAG3*, *SPP1*, *SEPP1*, *TREM2*, and *APOE*, supporting distinct transcriptional states within the myeloid compartment. (c) Dot plot comparing the same marker set across

myeloid subsets. Dot size indicates the percentage of expressing cells, and color indicates scaled average expression.

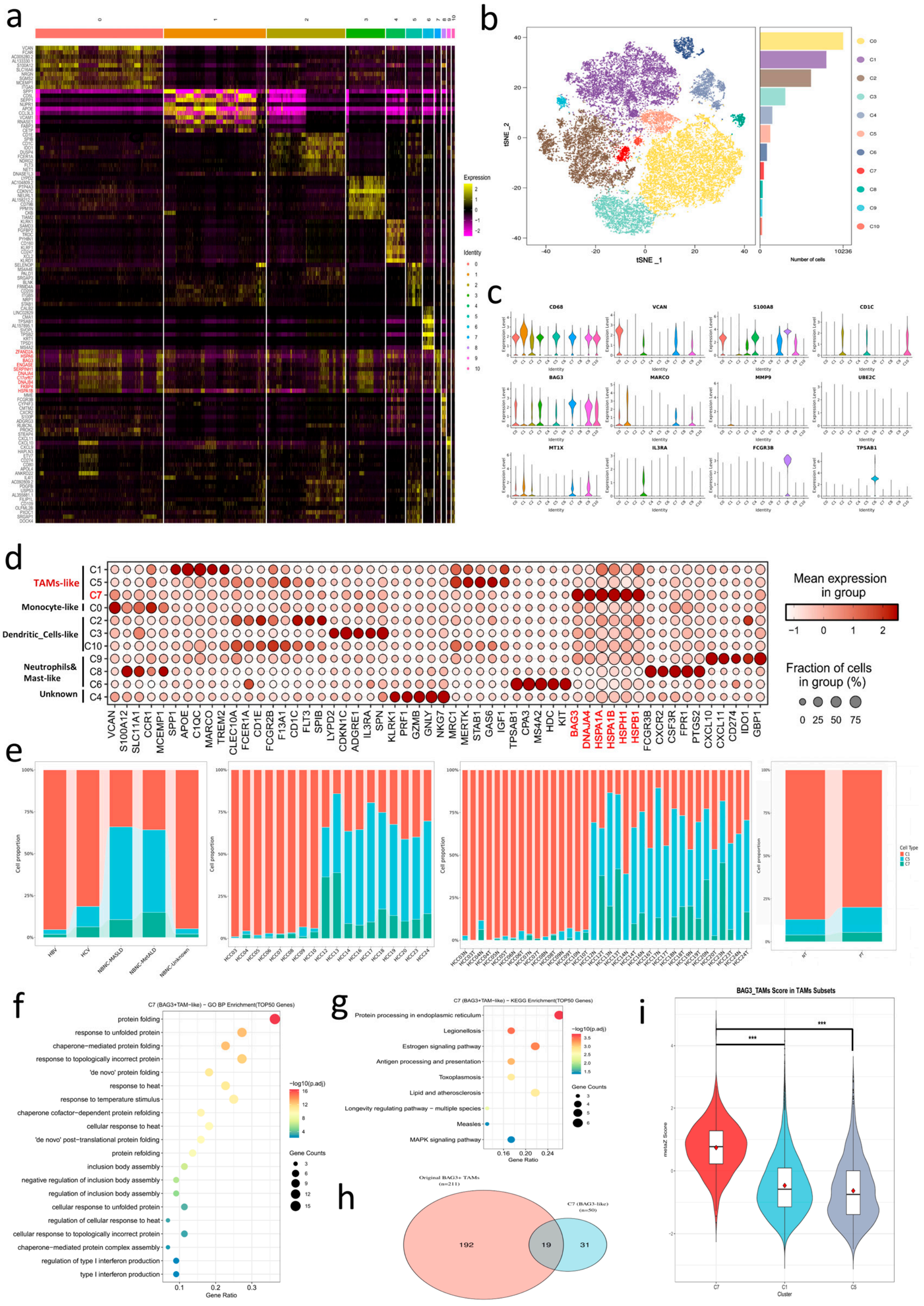

Supplementary Figure S4. Myeloid re-clustering and functional profiling in the integrated validation scRNA-seq dataset, highlighting the *BAG3*-enriched C7 cluster. (a) Heatmap of the top marker genes for each myeloid cluster following re-clustering (ranked by $\log_2$FC), highlighting C7-associated stress/chaperone markers

(e.g., *BAG3*, *HSPA1A*, *HSPB1*, and *DNAJB1*). **(b)** The t-SNE projection of myeloid clusters and bar plot of cell counts per cluster, highlighting C7. **(c)** Violin plots showing expression distributions of representative monocyte/macrophage markers (e.g., *BAG3*, *CD68*, *VCAN*, and *MRC1*) across clusters. **(d)** Dot plot summarizing marker expression across clusters (dot size indicates fraction of expressing cells; color indicates mean expression), with broad functional labels for major myeloid states. Key C7-associated stress/chaperone genes (e.g., *BAG3*, *HSPA1A*, *HSPB1*, and *DNAJB1*) are highlighted in red. **(e)** Compositional proportions of key TAM-like clusters (C1, C5, and C7) across etiology, patient, samples, and tissue site stratification. **(f)** GO-BP enrichment bubble plot for C7 marker genes (bubble size indicates gene ratio), highlighting proteostasis- and stress-related terms. **(g)** KEGG enrichment bubble plot for C7 marker genes (bubble size indicates gene ratio), highlighting antigen processing/presentation, lysosome, and MAPK signaling. **(h)** Venn diagram comparing C7 marker genes with reference TAM signatures ($\log_2 FC$ threshold as indicated), showing shared and C7-specific gene sets. **(i)** Comparison of $BAG3^+$ TAM scores (top-50 genes) across C1/C5/C7, with C7 showing the highest enrichment ($p < 0.001$).

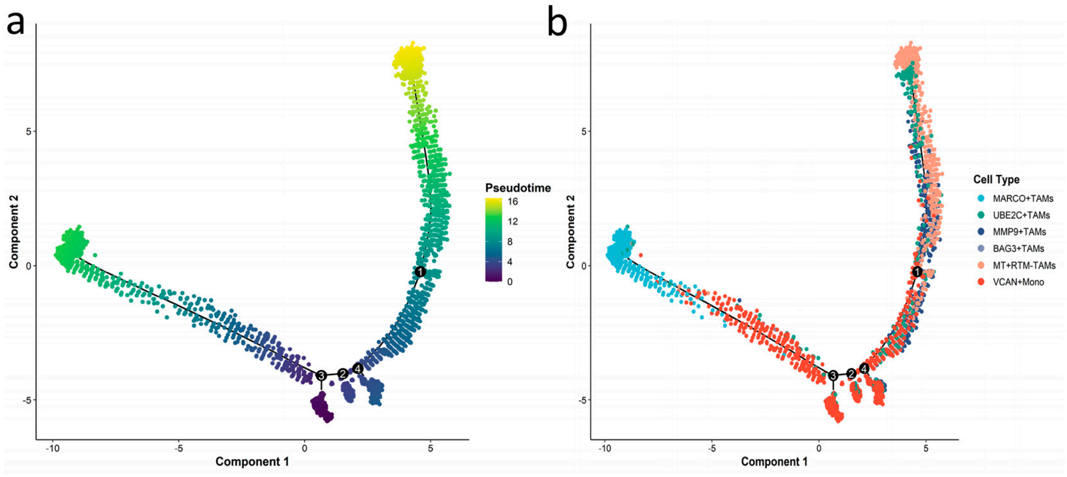

Supplementary Figure S5. Exploratory pseudotime analysis of non-HBV-derived TAM subsets. (a) Pseudotime trajectory of TAM subsets from non-HBV samples, including HCV and NBNC cases, colored by cell-type annotation. (b) The same trajectory colored by inferred pseudotime. This analysis was performed as a sensitivity analysis and should be interpreted cautiously because of etiologic heterogeneity and limited BAG3^{+} TAM representation in non-HBV samples.

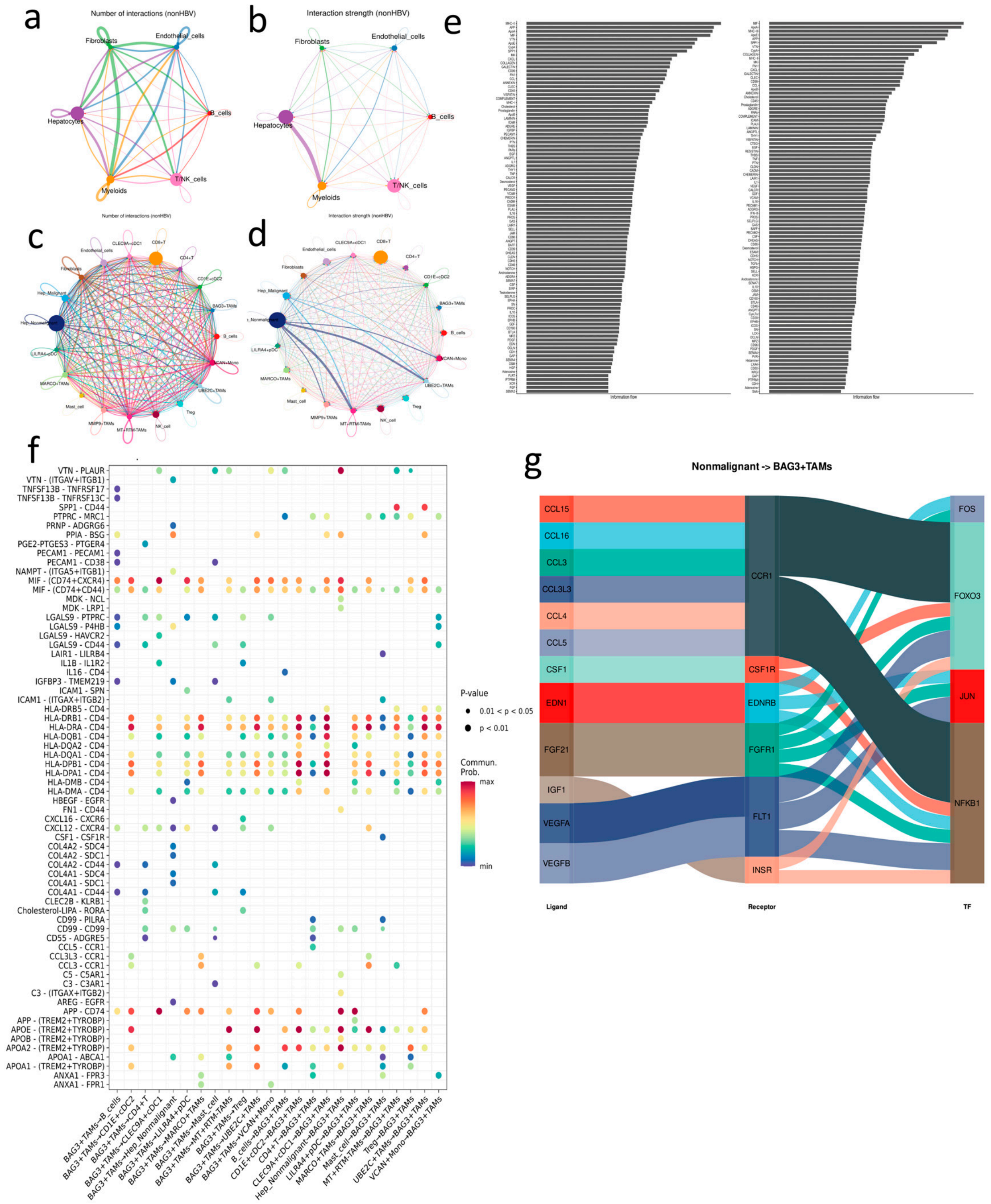

Supplementary Figure S6. Panoramic view of cell communication and the BAG3⁺ TAM-centered network. (a) Aggregated CellChat communication network among major cell types; edge width represents overall communication strength. **(b)** Information flow of representative signaling pathways, summarizing total incoming and outgoing strengths across cell types. **(c)** Subpopulation-level network emphasizing BAG3⁺ TAMs as sender cells. **(d)** Subpopulation-level network emphasizing BAG3⁺ TAMs as receiver cells; edge direction indicates signal flow from sender to receiver. **(e)** Ranked incoming and outgoing ligand–receptor interactions for BAG3⁺ TAMs under

HBV-related and non-HBV conditions. **(f)** Bubble plot of additional ligand–receptor interactions not shown in the main figure; rows represent ligand–receptor pairs and columns represent sender–receiver cell-type pairs (color indicates communication probability; dot size indicates significance). **(g)** Representative CellCall Sankey plot of inferred non-malignant hepatocyte-to-BAG3^{+} TAM ligand–receptor–transcription factor circuits in the HBV-derived analysis subset; ligands, receptors, and downstream transcription factors are shown from left to right, and edge width indicates inferred communication weight.

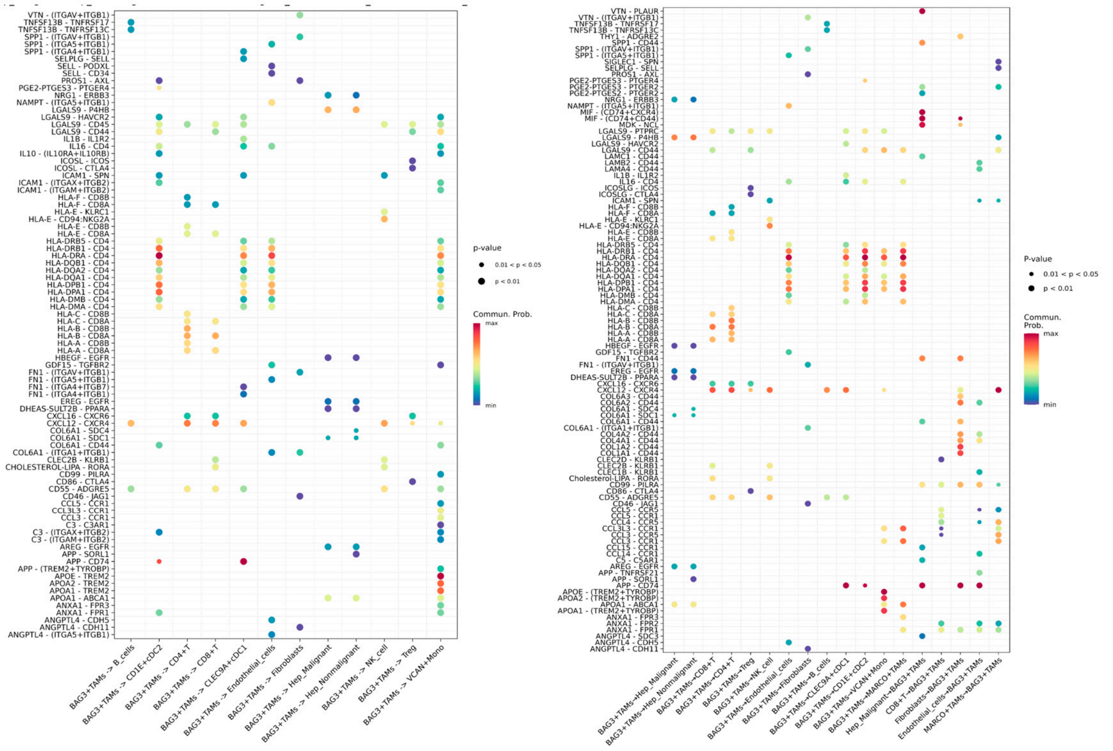

Supplementary Figure S7. Bubble plot of ligand-receptor interactions involving BAG3⁺ TAMs in the non-HBV condition. Bubble plot summarizing ligand–receptor pairs across sender–receiver cell-type pairs in non-HBV samples (color indicates communication probability; dot size indicates significance).

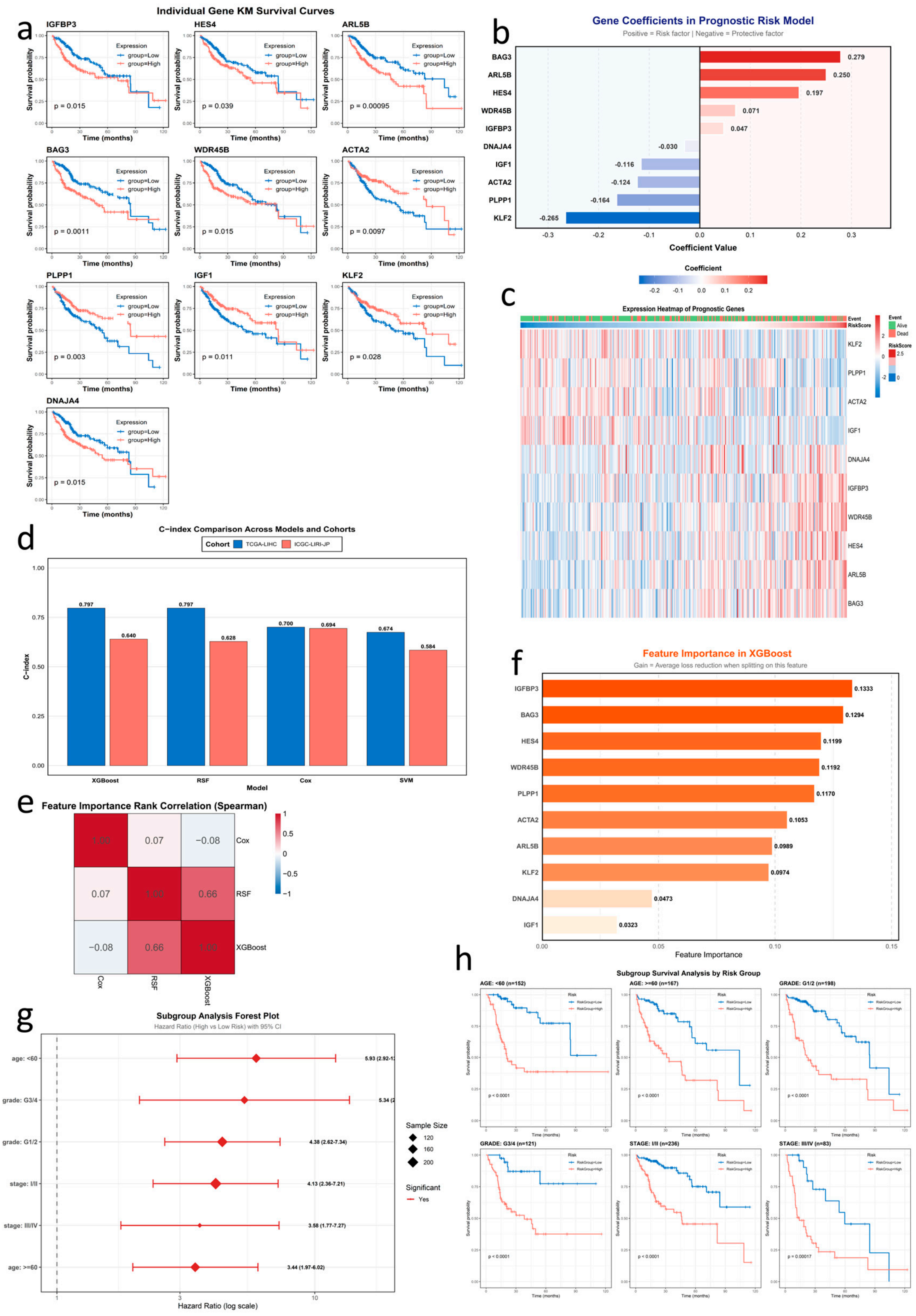

Supplementary Figure S8. Extended multi-model evaluation of the 10-gene prognostic signature in HCC. (a) Kaplan–Meier curves for each of the 10 genes in TCGA-LIHC, with log-rank p values. **(b)** Elastic-net Cox coefficients (α = 0.5) for the 10 genes, distinguishing positive and negative coefficients. **(c)** Heatmap of expression

for the 10 genes in TCGA-LIHC, with patients ordered by RS and genes ordered by coefficients; survival status and RS are annotated. **(d)** Bar plot comparing Harrell's C-index across four survival models (multivariable Cox, RSF, XGBoost, and SVM) in TCGA-LIHC and ICGC-LIRI-JP. **(e)** Correlation heatmap (Spearman's ρ) comparing gene-importance rankings derived from the multivariable Cox, RSF, and XGBoost models. **(f)** Horizontal bar plot showing XGBoost feature importance for each gene in the 10-gene signature. **(g)** Subgroup forest plot showing hazard ratios for the RS across clinical strata. **(h)** Kaplan–Meier curves comparing risk groups within age, grade, and AJCC-stage subgroups.

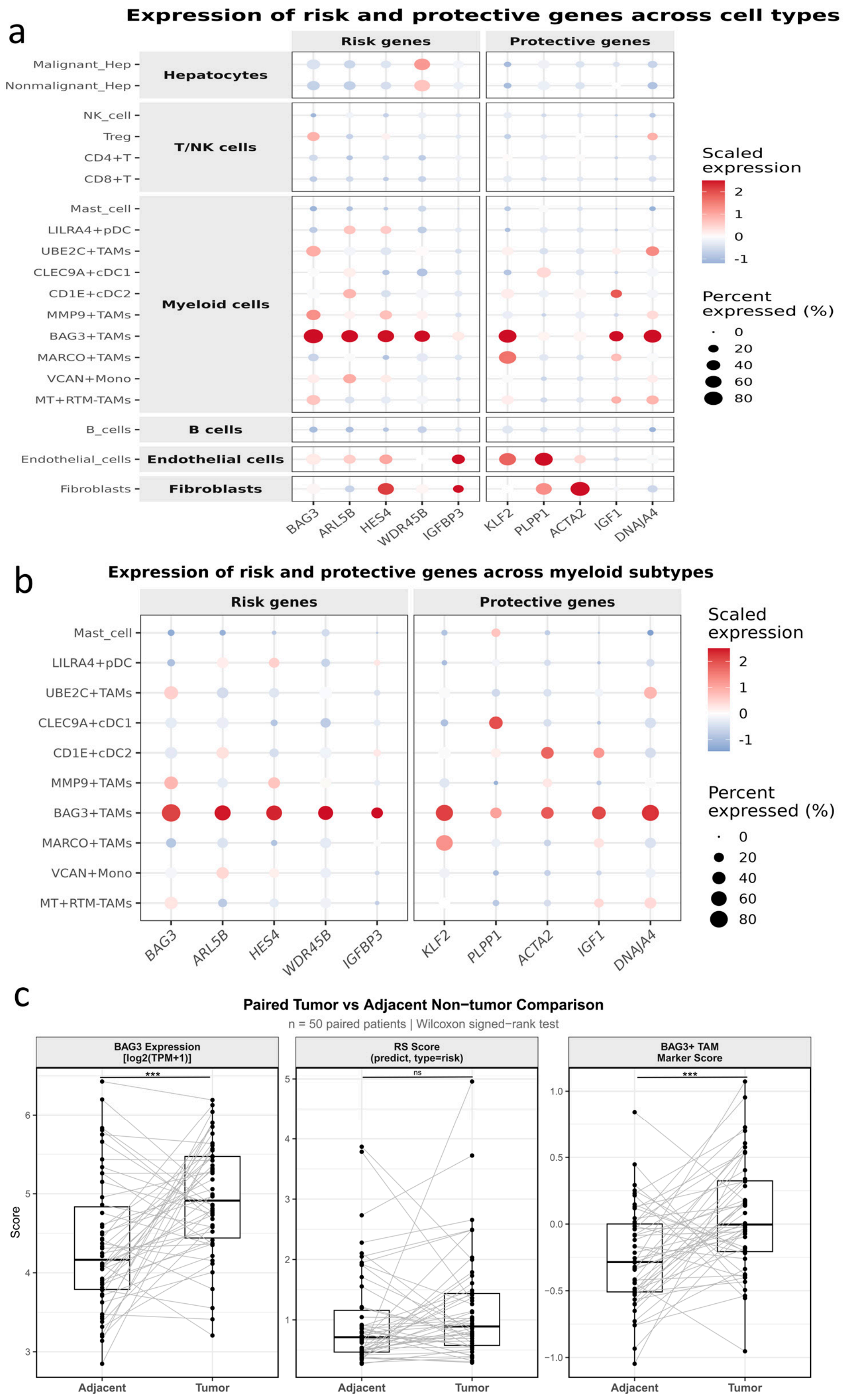

Supplementary Figure S9. Cell-type expression patterns of the final *BAG3*-associated prognostic signature genes. (a) Dot plot showing the expression of risk-associated and protective genes across major cell populations. **(b)** Dot plot showing the expression of the same genes across myeloid subsets. Dot size indicates the percentage

of expressing cells, and color indicates scaled average expression. **(c)** Paired tumor-adjacent comparison of *BAG3*-associated signals in 50 paired TCGA-LIHC bulk RNA-seq samples, including *BAG3* expression [$\log_2$(TPM+1)], the coefficient-weighted 10-gene RS formula score, and the BAG3$^+$ TAM marker-program score. Lines connect paired tumor and adjacent non-tumor samples from the same patient. P values were calculated using paired Wilcoxon signed-rank tests and adjusted using the Benjamini–Hochberg method across the three comparisons. ns, not significant; ***FDR < 0.001.

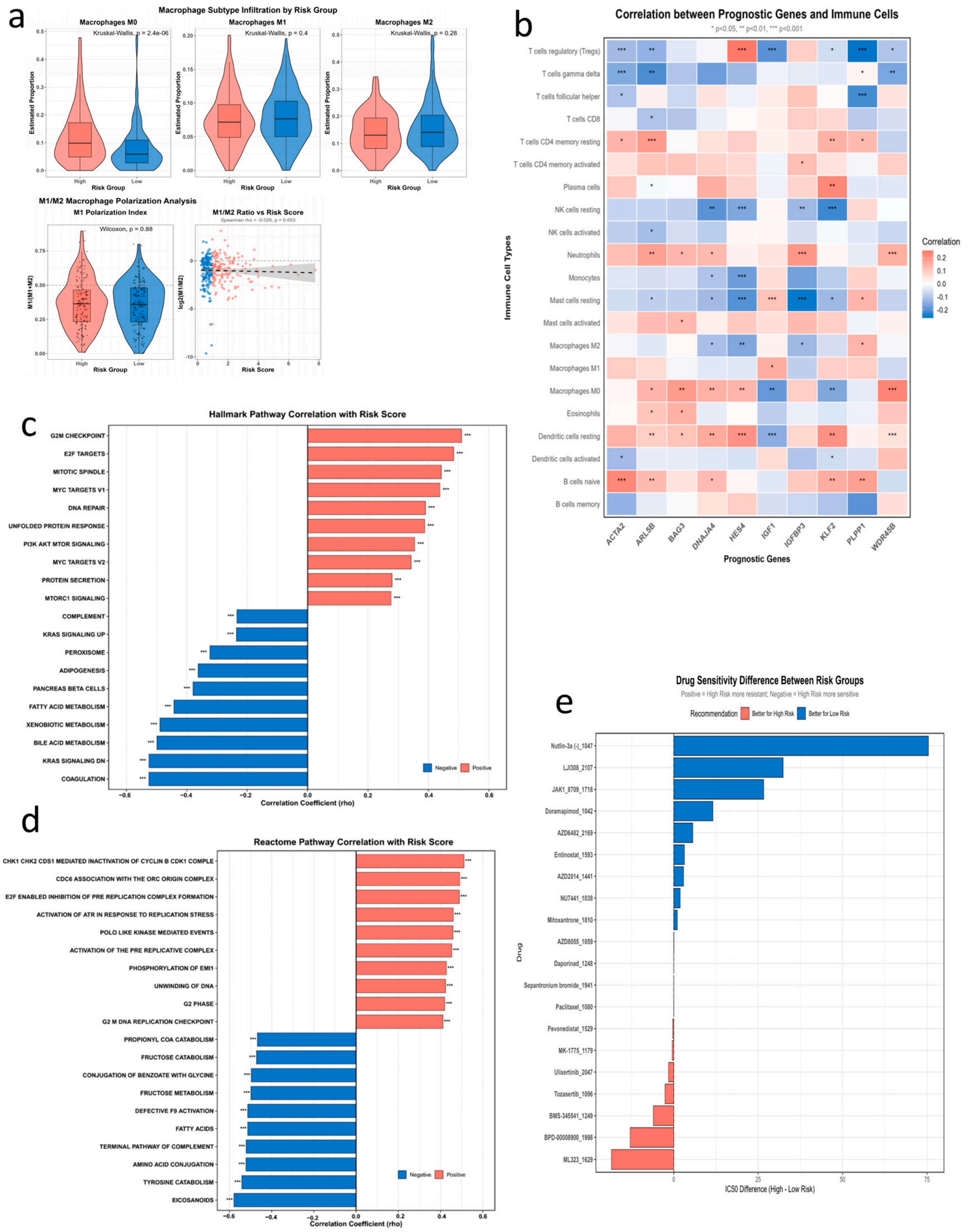

Supplementary Figure S10. Immune, pathway and drug response correlates of the *BAG3*-associated risk score. (a) Violin plots of CIBERSORT-inferred macrophage subsets (M0, M1, M2) and polarization metrics (M1 polarization index) stratified by risk group, showing enrichment of M0-like macrophages in the high-risk group ($p = 2.4 \times 10^{-6}$). **(b)** Spearman correlation heatmap between the 10 prognostic genes and 22

immune cell types estimated by CIBERSORT; asterisks indicate statistical significance. **(c)** Diverging horizontal bar plot of Spearman correlations between Hallmark pathway activity scores (ssGSEA) and the RS, showing the top positively and negatively correlated pathways. **(d)** Diverging horizontal bar plot of Spearman correlations between Reactome pathway activity scores (ssGSEA) and the risk score, showing the top positively and negatively correlated pathways. **(e)** Diverging horizontal bar plot of risk-group differences in predicted drug sensitivity (IC50) for the top differential compounds; positive values indicate higher predicted IC50 in the high-risk group. *$P < 0.05$, **$P < 0.01$, ***$P < 0.001$.

Supplementary Table S1. Overview of discovery and integrated validation HCC scRNA-seq datasets.

Dataset	Patient ID	Gender	Age (in years)	HCC Etiology	Samples	Etiology of liver disease					GEO Accession
						Virus	Alcohol	Obesity	MASH	SLD	
Discovery dataset	HCC01	Male	66	HBV-HCC	HCC01T	HBV	NA	NA	NA	NA	GSE149614
	HCC02	Male	65	HBV-HCC	HCC02T	HBV	NA	NA	NA	NA	GSE149614
	HCC03	Male	66	HCV-HCC	HCC03N, HCC03T	HCV	NA	NA	NA	NA	GSE149614
	HCC04	Male	60	HCV-HCC	HCC04N, HCC04T	HCV	NA	NA	NA	NA	GSE149614
	HCC05	Male	65	NBNC-HCC （Unknown）	HCC05N, HCC05T	No	NA	NA	NA	NA	GSE149614
	HCC06	Female	64	HBV-HCC	HCC06N, HCC06T	HBV	NA	NA	NA	NA	GSE149614
	HCC07	Male	48	NBNC-HCC （Unknown）	HCC07N, HCC07P, HCC07T	No	NA	NA	NA	NA	GSE149614
	HCC08	Male	64	NBNC-HCC （Unknown）	HCC08N, HCC08P, HCC08T	No	NA	NA	NA	NA	GSE149614
	HCC09	Male	48	HBV-HCC	HCC09N, HCC09T	HBV	NA	NA	NA	NA	GSE149614
	HCC10	Male	53	HBV-HCC	HCC10L, HCC10N, HCC10T	HBV	NA	NA	NA	NA	GSE149614
	HCC03	Male	66	HCV-HCC	HCC03N,	HCV	NA	NA	NA	NA	GSE149614

					HCC03T						
Integrated validation dataset	HCC04	Male	60	HCV-HCC	HCC04N, HCC04T	HCV	NA	NA	NA	NA	GSE149614
	HCC05	Male	65	NBNC-HCC（Unknown）	HCC05N, HCC05T	No	NA	NA	NA	NA	GSE149614
	HCC06	Female	64	HBV-HCC	HCC06N, HCC06T	HBV	NA	NA	NA	NA	GSE149614
	HCC07	Male	48	NBNC-HCC（Unknown）	HCC07N, HCC07T	No	NA	NA	NA	NA	GSE149614
	HCC08	Male	64	NBNC-HCC（Unknown）	HCC08N, HCC08T	No	NA	NA	NA	NA	GSE149614
	HCC09	Male	48	HBV-HCC	HCC09N, HCC09T	HBV	NA	NA	NA	NA	GSE149614
	HCC10	Male	53	HBV-HCC	HCC10N, HCC10T	HBV	NA	NA	NA	NA	GSE149614
	HCC12	Female	68	HCV-HCC	HCC12N, HCC12T	HCV	No	Yes	No	No	GSE245906
	HCC13	Male	63	NBNC-MetALD-HCC	HCC13N, HCC13T	No	Yes	Yes	Yes	Yes	GSE245906
	HCC14	Male	62	NBNC-MASLD-HCC	HCC14N, HCC14T	No	No	No	No	Yes	GSE245906
	HCC16	Male	85	HCV-HCC	HCC16N,	HCV	No	No	No	No	GSE245906

				HCC16T						
HCC17	Male	78	NBNC-HCC (Unknown)	HCC17N, HCC17T	No	No	No	No	No	GSE245906
HCC18	Male	78	NBNC-MASLD-HCC	HCC18N, HCC18T	No	No	No	No	Yes	GSE245906
HCC19	Male	60	NBNC-MetALD-HCC	HCC19N, HCC19T	No	Yes	Yes	No	Yes	GSE245906
HCC20	Male	74	NBNC-MetALD-HCC	HCC20N, HCC20T	No	Yes	No	No	Yes	GSE245906
HCC23	Male	70	NBNC-MetALD-HCC	HCC23N, HCC23T	No	Yes	Yes	Yes	Yes	GSE245906
HCC24	Male	69	HCV-HCC	HCC24N, HCC24T	HCV	No	Yes	No	No	GSE245906

Abbreviations: HCC, hepatocellular carcinoma; HBV, hepatitis B virus; HCV, hepatitis C virus; NBNC, non-HBV/non-HCV (non-viral) cases; scRNA-seq, single-cell RNA sequencing; GEO, Gene Expression Omnibus; SLD, steatotic liver disease (i.e., hepatic steatosis); MASLD, metabolic dysfunction–associated steatotic liver disease; MASH, metabolic dysfunction–associated steatohepatitis (the inflammatory subtype of MASLD); MetALD, metabolic dysfunction–associated steatotic liver disease with coexisting alcohol exposure. Unknown (within NBNC-HCC) indicates non-viral cases not further classified into MASLD/MASH/MetALD based on the available metadata. NA, not available (information not provided in the original studies/metadata). Detailed non-viral etiology annotations (alcohol/obesity/MASH/SLD) were not consistently available across the source studies; unavailable fields are shown as NA. Definitions and nomenclature for SLD/MASLD/MASH/MetALD follow the recent multi-society consensus on steatotic liver disease terminology. Age indicates age at surgery. Sample suffixes denote tissue origin: N, peri-tumour (adjacent non-tumour) tissue; T, primary tumour tissue; P, portal vein thrombus; L, lymph-node metastasis.

Supplementary Table S2. Spatial transcriptomic QC metrics for four HCC samples (GSE238264).

Sample_ID (this study)	Sample_ID (original)	Group	Total_spots	Tissue_spots	Total_genes	Median_genes_ per_spot	Median_UMI	Median_mt_percent(%)
ST01-R	hcc1-R	Responder	3006	3006	36601	1360	2920	3.68
ST02-R	hcc3-R	Responder	2170	2170	36601	1012	2230	12.93
ST03-R	hcc4-R	Responder	3002	3002	36601	3874	11350	5.74
ST04-NR	hcc6-NR	Non-responder	2575	2575	36601	4512	22734	4.60

Abbreviations: R, responder (≥90% tumor necrosis) after neoadjuvant cabozantinib plus nivolumab; NR, non-responder (<90% tumor necrosis); UMI, unique molecular identifier; mt%, mitochondrial transcript percentage. Total_spots and Tissue_spots denote the total number of sequenced spots and spots retained as tissue, respectively. Median_genes_per_spot, Median_UMI, and Median_mt_percent (mt%) are calculated per spot. Sample_ID (original) corresponds to the identifier reported in the source study (GEO: GSE238264) for traceability.

Supplementary Table S3. Differential transcription factor activity in high and low BAG3⁺ TAM groups across multiple quantile thresholds.

Patient	Quantile	TF	N_targets_available	N_High	N_Low	Delta_Mean	Delta_Median	Wilcox_p	Cliffs_delta	Wilcox_p_adj
ST04-NR	75	AP1	110	128	381	0.343	0.279	3.29E-03	0.174	4.94E-03
		NFKB1	50	128	381	0.398	0.386	4.63E-05	0.241	1.39E-04
		EGR1	50	128	381	0.382	0.406	1.39E-05	0.257	1.04E-04
		FOXO3	21	128	381	0.277	0.104	1.46E-02	0.144	1.83E-02
		RB1	30	128	381	-0.079	-0.088	3.50E-01	-0.055	4.03E-01
	80	AP1	110	102	407	0.352	0.264	9.51E-03	0.166	1.30E-02
		NFKB1	50	102	407	0.414	0.396	8.05E-05	0.252	2.01E-04
		EGR1	50	102	407	0.416	0.399	2.50E-05	0.270	1.25E-04
		FOXO3	21	102	407	0.333	0.170	2.62E-03	0.193	4.36E-03
		RB1	30	102	407	0.010	0.041	9.02E-01	0.008	9.20E-01
	85	AP1	110	77	432	0.445	0.441	1.30E-03	0.230	2.43E-03
		NFKB1	50	77	432	0.553	0.494	5.62E-06	0.325	8.42E-05
		EGR1	50	77	432	0.452	0.396	4.05E-05	0.293	1.39E-04
		FOXO3	21	77	432	0.416	0.225	8.71E-04	0.238	1.87E-03
		RB1	30	77	432	0.019	0.065	9.20E-01	0.007	9.20E-01
ST03-R	75	AP1	110	87	261	0.275	0.118	2.78E-02	0.158	4.94E-02
		NFKB1	50	87	261	0.366	0.317	3.17E-03	0.211	9.51E-03
		EGR1	50	87	261	0.382	0.392	1.83E-03	0.223	6.88E-03
		FOXO3	21	87	261	0.047	0.113	6.04E-01	0.037	6.04E-01
		RB1	30	87	261	-0.289	-0.249	3.12E-02	-0.154	4.94E-02
	80	AP1	110	70	278	0.377	0.236	6.39E-03	0.211	1.60E-02
		NFKB1	50	70	278	0.457	0.413	7.26E-04	0.261	6.52E-03
		EGR1	50	70	278	0.444	0.471	1.14E-03	0.252	6.52E-03

		FOXO3	21	70	278	0.151	0.208	1.82E-01	0.103	2.28E-01
		RB1	30	70	278	-0.194	-0.165	2.14E-01	-0.096	2.47E-01
		AP1	110	53	295	0.355	0.136	3.30E-02	0.184	4.94E-02
		NFKB1	50	53	295	0.494	0.464	1.30E-03	0.277	6.52E-03
	85	EGR1	50	53	295	0.416	0.465	8.86E-03	0.226	1.90E-02
		FOXO3	21	53	295	0.159	0.111	2.89E-01	0.092	3.10E-01
		RB1	30	53	295	-0.227	-0.214	1.69E-01	-0.119	2.28E-01
		AP1	109	146	437	0.360	0.314	5.37E-04	0.191	4.64E-03
		NFKB1	50	146	437	0.227	0.164	5.03E-02	0.108	8.38E-02
	75	EGR1	50	146	437	0.342	0.339	9.66E-04	0.182	4.83E-03
		FOXO3	21	146	437	0.227	0.184	2.10E-02	0.127	5.25E-02
		RB1	30	146	437	0.147	0.141	1.70E-01	0.076	1.96E-01
		AP1	109	117	466	0.258	0.208	2.83E-02	0.131	5.37E-02
		NFKB1	50	117	466	0.141	0.012	3.33E-01	0.058	3.33E-01
ST02-R	80	EGR1	50	117	466	0.358	0.307	1.91E-03	0.185	7.17E-03
		FOXO3	21	117	466	0.209	0.174	6.40E-02	0.111	9.59E-02
		RB1	30	117	466	0.126	0.163	3.05E-01	0.061	3.27E-01
		AP1	109	88	495	0.356	0.326	5.51E-03	0.186	1.65E-02
		NFKB1	50	88	495	0.232	0.197	7.31E-02	0.120	9.97E-02
	85	EGR1	50	88	495	0.448	0.368	6.18E-04	0.229	4.64E-03
		FOXO3	21	88	495	0.279	0.211	2.86E-02	0.146	5.37E-02
		RB1	30	88	495	0.220	0.222	8.11E-02	0.117	1.01E-01
		AP1	109	122	363	0.282	0.267	7.51E-03	0.162	1.41E-02
ST01-R	75	NFKB1	50	122	363	0.315	0.284	2.52E-03	0.183	1.41E-02
		EGR1	50	122	363	0.255	0.369	4.25E-03	0.173	1.41E-02

	FOXO3	21	122	363	0.201	0.235	3.41E-02	0.128	4.26E-02
	RB1	30	122	363	-0.023	0.108	8.39E-01	0.012	8.77E-01
	AP1	109	97	388	0.308	0.272	5.64E-03	0.182	1.41E-02
	NFKB1	50	97	388	0.314	0.203	6.57E-03	0.178	1.41E-02
80	EGR1	50	97	388	0.259	0.420	7.12E-03	0.177	1.41E-02
	FOXO3	21	97	388	0.256	0.310	1.12E-02	0.166	1.86E-02
	RB1	30	97	388	-0.117	-0.019	4.14E-01	-0.054	4.77E-01
	AP1	109	73	412	0.348	0.335	5.60E-03	0.203	1.41E-02
	NFKB1	50	73	412	0.432	0.338	1.18E-03	0.238	1.41E-02
85	EGR1	50	73	412	0.272	0.543	1.29E-02	0.182	1.94E-02
	FOXO3	21	73	412	0.254	0.305	2.76E-02	0.162	3.76E-02
	RB1	30	73	412	-0.079	0.094	8.77E-01	-0.011	8.77E-01

Abbreviations: TF, transcription factor; FDR, false discovery rate. Notes: Patient indicates the tissue section (e.g., ST04-NR). Quantile denotes the upper-quantile cutoff used to define BAG3$^+$ TAM metaZ high and low regions. N_High and N_Low indicate the numbers of spots in the high- and low-score groups, respectively. N_targets_available is the number of TF target genes available and used for TF activity inference. Delta_Mean and Delta_Median represent TF activity differences between groups (high and low) based on mean and median values, respectively. Wilcox_p and Wilcox_p_adj are two-sided Wilcoxon rank-sum p values before and after FDR correction. Cliffs_delta denotes Cliff’s δ effect size (positive values indicate higher TF activity in BAG3$^+$ TAM high regions).

Supplementary Table S4. Prognostic association of 13 BAG3⁺ TAM marker genes identified by univariable Cox analysis (TCGA-LIHC).

Gene	Coefficient	SE	Z	P_value	HR	SE(log HR)	Z (HR)	P_value (HR)	95% CI (HR), Lower	95% CI (HR), Upper
ARL5B	0.649	0.200	3.251	0.001	1.913	0.382	2.392	0.017	1.294	2.829
BAG3	0.638	0.198	3.215	0.001	1.893	0.375	2.377	0.017	1.283	2.792
PLPP1	-0.582	0.199	-2.930	0.003	0.559	0.111	-3.975	0.000	0.379	0.825
ACTA2	-0.506	0.198	-2.558	0.011	0.603	0.119	-3.329	0.001	0.409	0.889
IGF1	-0.492	0.196	-2.505	0.012	0.611	0.120	-3.237	0.001	0.416	0.898
IGFBP3	0.476	0.196	2.421	0.016	1.609	0.316	1.927	0.054	1.095	2.365
DNAJA4	0.480	0.199	2.411	0.016	1.616	0.322	1.915	0.056	1.094	2.387
WDR45B	0.470	0.196	2.402	0.016	1.600	0.313	1.917	0.055	1.090	2.348

KLF2	-0.428	0.196	-2.177	0.030	0.652	0.128	-2.716	0.007	0.444	0.958
SPARCL1	-0.412	0.197	-2.095	0.036	0.663	0.130	-2.592	0.010	0.451	0.974
HES4	0.402	0.196	2.051	0.040	1.495	0.293	1.689	0.091	1.018	2.194
PLVAP	-0.390	0.196	-1.994	0.046	0.677	0.133	-2.439	0.015	0.461	0.993
SERPINC1	-0.388	0.198	-1.961	0.050	0.679	0.134	-2.396	0.017	0.461	1.000

Note: Coefficient is the Cox regression coefficient (log hazard ratio), SE is its standard error, and HR is the hazard ratio (HR = exp(Coefficient)) with its 95% CI. Reported P values are nominal (no multiple-testing correction).

Supplementary Table S5. Feature importance comparison across three survival prediction models.

Gene	Importance	Model	Importance_Normalization	Rank
IGFBP3	0.051	Cox	0.000	10
HES4	0.261	Cox	0.701	4
ARL5B	0.314	Cox	0.879	3
BAG3	0.350	Cox	1.000	1
WDR45B	0.068	Cox	0.057	9
ACTA2	0.148	Cox	0.324	7
PLPP1	0.194	Cox	0.478	5
IGF1	0.154	Cox	0.343	6
KLF2	0.328	Cox	0.925	2
DNAJA4	0.106	Cox	0.183	8
IGFBP3	0.037	RSF	0.754	5
HES4	0.044	RSF	0.931	3
ARL5B	0.045	RSF	0.953	2
BAG3	0.040	RSF	0.838	4
WDR45B	0.046	RSF	1.000	1
ACTA2	0.016	RSF	0.208	7
PLPP1	0.030	RSF	0.560	6

IGF1	0.009	RSF	0.000	10
KLF2	0.016	RSF	0.192	8
DNAJA4	0.016	RSF	0.189	9
IGFBP3	0.133	XGBoost	1.000	1
BAG3	0.129	XGBoost	0.962	2
HES4	0.120	XGBoost	0.867	3
WDR45B	0.119	XGBoost	0.860	4
PLPP1	0.117	XGBoost	0.839	5
ACTA2	0.105	XGBoost	0.723	6
ARL5B	0.099	XGBoost	0.660	7
KLF2	0.097	XGBoost	0.645	8
DNAJA4	0.047	XGBoost	0.149	9
IGF1	0.032	XGBoost	0.000	10

Note: Importance refers to model-specific feature importance (Cox: absolute coefficient magnitude; RSF/XGBoost: model-derived importance scores). Importance_Normalization is a within-model rescaling to [0,1] for comparability of ranks, and Rank is ordered from highest to lowest importance.

Supplementary Table S6. Sensitivity analysis of the association between the final Cox-based RS and overall survival after adjustment for available etiology/risk-factor category.

Model	RS variable	Adjusted covariates	N	Events	HR	95% CI	P value
Original multivariable Cox	High vs low RS	age group, grade group, AJCC stage group	319	107	2.992	1.941–4.613	6.88×10^{-7}
Etiology-adjusted Cox	High vs low RS	age group, grade group, AJCC stage group, etiology/risk-factor category	319	107	2.929	1.878–4.570	2.17×10^{-6}

Note: HRs are shown only for the RS variable because this sensitivity analysis was designed to evaluate whether the association between the final Cox-based RS and overall survival remained after additional adjustment for available etiology/risk-factor category. Age, tumor grade, and AJCC stage were dichotomized as <60 versus ≥60, G1/G2 versus G3/G4, and stage I/II versus III/IV, respectively. Etiology/risk-factor categories were derived from the TCGA-LIHC field "history_hepato_carcinoma_risk_factors" and grouped as HBV, HCV, and NBNC/Other. NBNC/Other included non-HBV/non-HCV cases, including alcohol-related, NAFLD-related, no documented primary risk factor, other, and unavailable/blank annotations. Etiology/risk-factor categories were included as adjustment covariates only, and category-specific etiology coefficients were not interpreted mechanistically. RS, risk score; HR, hazard ratio; CI, confidence interval; AJCC, American Joint Committee on Cancer; HBV, hepatitis B virus; HCV, hepatitis C virus; NBNC, non-HBV/non-HCV.
